# The impact of life events and transitions on physical activity: A scoping review

**DOI:** 10.1371/journal.pone.0234794

**Published:** 2020-06-22

**Authors:** Hannes Gropper, Jannika M. John, Gorden Sudeck, Ansgar Thiel

**Affiliations:** 1 Institute of Sports Science, Eberhard Karls University Tübingen, Tübingen, Germany; 2 Interfaculty Research Institute for Sport and Physical Activity, Eberhard Karls University Tübingen, Tübingen, Germany; University of Ottawa Heart Institute, CANADA

## Abstract

**Background:**

Physical activity (PA) is a fluctuating behavior and prone to change across the life course. Changes in PA may be particularly due to the experience of life events and transitions. For well-timed and successful PA interventions, it is important to understand when and why individuals take up or terminate PA.

**Objectives:**

This scoping review aims to examine the extent, range, and nature of research on the impact of life events and transitions on PA and to summarize key findings.

**Methods:**

A systematic literature search was conducted in PubMed, PsycINFO, PsycARTICLES, SPORTDiscus, and Web of Science. Articles were included if they had been published in peer-reviewed journals between 1998 and 2020 and assessed the impact of at least one life event or transition on PA.

**Results:**

107 studies that assessed 72 distinct life events and transitions were included and summarized in ten categories. Events and transitions that are primarily associated with decreases in PA were starting cohabitation, getting married, pregnancy, evolving parenthood, and the transitions from kindergarten to primary school, from primary to secondary school, and from high school to college or into the labor market. Retirement was associated with increases in PA; yet, long-term trajectories across retirement indicated a subsequent drop in activity levels. Divorce was associated with no changes in PA. No trends could be identified for changing work conditions, quitting or losing a job, starting a new relationship, widowhood, moving, and diagnosis of illness.

**Conclusion:**

Life events and transitions can be conceptualized as natural interventions that occur across the life course and that are oftentimes associated with changes in PA behavior. Our study indicates that, despite some emerging trends, similar events do not necessarily have similar impacts on PA across individuals. It also shows that the research landscape is characterized by a lack of conceptual clarity and by disparate methodologies, making it difficult to synthesize results across studies.

## Introduction

Time and again, research has shown that regular physical activity (PA) is associated with physical [1, 2], as well as mental and social [3, 4] health benefits. Thus, PA is considered “a ‘best buy’ for public health” [5]. However, PA is a highly complex and multidimensional behavioral phenomenon [6], which is prone to change across the life course [7]. Despite being a significant promoter of health and well-being, regular PA tends to decrease over the life course [8]. Total PA appears to decline particularly during adolescence and the transition to young adulthood [9]. Additionally, Shaw et al. [10] found “steady declines in leisure-time physical activity, beginning in midlife and growing steeper at progressively older ages” (p.763). Contrary to these findings, some longitudinal studies have also identified groups that remain at a given activity level or even increase their amount of PA [11–13].

These results suggest that PA is a *fluctuating* behavior that is not stable over time [14], marked by lapses and relapses [15], and shows a more pronounced trend towards declining than towards stable or increasing levels. This is in line with research arguing that PA patterns track at rather low to moderate levels across the life course [16, 17]. Considering both long-term and short-term health benefits of regular PA [5], it is crucial to understand when and why people take up or terminate PA. Discontinuity, and associated behavioral lapses and relapses, might be triggered particularly by the experience of life events and transitions that occur across the life course. Telama [17] summarizes that “because many transitions and life-changing events experienced during the course of life influence physical activity, the level of tracking of physical activity is likely to vary at different phases of life” (p. 187).

Previously, two reviews have *explicitly* focused on the impact of various life-change events on PA. Allender et al. [18] identified five general life changes that primarily account for drop out from PA, namely, changes in employment status, residence, physical status, relationships, and family structure. In addition, Engberg et al. [19] found evidence that major life events (e.g. the transition to university, beginning to work, cohabitation and marriage, pregnancy and childbirth, divorce, widowhood, and retirement) may have both positive and negative effects on PA, depending among others on age and gender. Recently, an umbrella review on the behavioral determinants of PA across the life course has shown negative associations between PA and the transition to university or emerging parenthood [20], while another review has argued that major life events, life transitions, and the experience of trauma can trigger stress, which in turn impairs efforts to be physically active [21]. In addition, several reviews have focused on particular life events and transitions. These previous reviews have suggested that the transition from primary to secondary school is associated with decreasing levels of total PA [22]. Moreover, leaving high school [23] and evolving parenthood [24–26] are linked to declines in PA, while retirement is linked to increases especially in leisure-time PA and exercise [27].

These reviews have contributed to the understanding of the impact of life events on PA and have shown that research on this topic is characterized by fragmentation and great heterogeneity of study designs and methods. Despite these achievements, we see three shortcomings in the recent review activity urging for a more comprehensive and up-to-date approach: First, the last review dealing *explicitly* with the question as to how various life events and PA relate to each other has been conducted in January 2011 [19] leaving the last nine years of research unmapped. Second, both Allender et al. [18] and Engberg et al. [19] searched only one (PubMed) and two databases (PubMed/MEDLINE and PsycINFO), respectively. Third, a theoretical underpinning of the search strategies and conceptual clarity in terminology is often missing.

Our aim is to examine the extent, range, and nature of previous research activity on the impact of life events and transitions on PA, to compile key findings, to identify research gaps, and, therefore, to provide an extension to the previous reviews [28, 29]. In particular, we aim to address the following research question that guided our process: How do life events and transitions impact PA behavior across the life course? We chose a scoping approach (i) to map the landscape of previous research activity in a field that is quite heterogeneous and fragmented and (ii) to systematically summarize key findings.

## Methods

In order to increase methodological rigor, the procedure of this scoping review adhered to the Preferred Reporting Items for Systematic Reviews and Meta-Analyses extension for Scoping Reviews (PRISMA-ScR) Checklist [30]. Due to a lack of conceptual and terminological clarity, in the following section we first explicate some theoretical considerations that have built the basis for our search strategy before we describe our methodological procedure in more detail.

### Theoretical and terminological considerations

We conceptualize life events from a developmental perspective arguing that they can be construed as *natural interventions* that occur across the life course and that may account for ontogenetic change, growth, and development [31]. According to the working definition put forward by Luhmann et al. [32], life events “mark the beginning or the end of a specific status. A status is a nominal variable with at least two levels” (p. 4). For example, marital status (i.e. single, married, divorced, or widowed) might change due to the life events of meeting a partner, marriage, divorce, or the death of a spouse.

Life events are thus singular occurrences that lead to a shift from one status to another. In this regard, they may *trigger* periods of biological, psychological, and/or social adaptation and readjustment and lead to behavioral changes. In the following, we term such adjustment periods *transitions* as they incorporate the processual character and temporality of life events and describe their anticipation (depending on the foreseeability of an event), history, and aftermath [33]. Both life events and transitions are interconnected and therefore two sides of the same experience, yet different in their temporal nature. Minor every-day events (e.g. daily hassles), slow transitions without clearly identifiable life events (e.g. aging, puberty), and non-events (e.g. *not* having a child) are excluded from our conceptualization of the terms life event and transition.

Life events and transitions may mark turning points in (habitual) behavioral patterns [31], such as PA. For our review, we employ a broad concept of PA defining it as any bodily movement produced by skeletal muscle resulting in energy expenditure [34]. PA therefore occurs across various domains and contexts such as leisure-time PA (including sport participation and exercise), occupational PA, active commuting, daily activities, or domestic activities and at different intensities such as light, moderate, vigorous, or moderate-to-vigorous. Table 1 summarizes the key concepts that underpin our review.

**Table 1:** Theoretical concepts.

Concept	Definition
**Life event**	In reference to the MeSH term definition for life-change events, yet with some adjustment, life events are conceptualized as singular occurrences (including biological, psychological, social, and environmental), which mark a change in status and therefore require (re)adjustment and effect a change in an individual’s pattern of living [31, 32, 35].
**Transition**	Transitions are conceptualized as status passages that temporally exceed the duration of life events. The duration of a transition varies as a function of the degree of (re)adjustment, which is necessary to adapt to a new status. Generally, life events, which imply a change in status, may trigger a transition or occur within the transitioning process itself (depending on an event’s foreseeability) [36, 37].
**Physical activity**	Physical activity is generally defined as any bodily movement produced by skeletal muscles resulting in energy expenditure [34] and may occur across various domains and contexts and at different intensities.

### Literature search

A broad, yet systematic, search strategy was developed to search the following databases: PubMed (including MEDLINE), PsycINFO, PsycARTICLES, SPORTDiscus, and Web of Science. Search terms were derived from our theoretical and terminological considerations and in accordance with the terms employed in previous reviews [18, 19]. Table 2 displays the search strategy, with #3 yielding the initial results from which studies where then selected. No limits were used.

**Table 2:** Search terms for the literature research.

Set	Search Terms
**#1**	“life event” OR “life events” OR “life change event” OR “life change events” OR “life-change event” OR “life-change events” OR “life changing event” OR “life changing events” OR “life-changing event” OR “life-changing events” OR “life experience” OR “life experiences” OR “life change” OR “life changes” OR “life-change” OR “life-changes” OR transition*
**#2**	“physical activity” OR exercis* OR “sport” OR “sports”
**#3**	#1 AND #2

The databases were searched on August 17, 2018. The search was updated on January, 27 2020. The updated information was combined with the results from the initial search. Results were exported into EndNote Citation Manager and duplicates were removed. Then, two researchers independently screened titles and abstracts for eligibility. Disagreements were resolved through a process of critical debate. If consensus could not be reached, a third researcher was consulted. Full-texts were assessed by a single researcher, who also extracted data from the articles. When in doubt, the inclusion of full-texts was discussed with a second researcher until consensus was reached. Moreover, included full-texts were used for cross-referencing.

### Inclusion and exclusion criteria

Papers were included if they (i) focused on life events and/or transitions in accordance with our theoretical conceptualizations; (ii) reported data on PA, which was assessed either objectively (e.g. via accelerometer or pedometer) or through self-report (e.g. via questionnaires or interviews); (iii) were either of prospective longitudinal or retrospective design in order to compare PA behavior before and after a life event or transition; (iv) assessed healthy populations, except for when transitions into disease were analyzed; (v) were published in English or German; (vi) were published in peer-reviewed journals between 1998 and 2020; and (vii) were available as full-texts.

Papers were excluded if they (i) were non-empirical (e.g. other reviews, editorials, comments, essays, abstracts, conference submissions, etc.), dissertations, or intervention studies (including randomized controlled trials); (ii) focused only on physical education or sedentary behavior; (iii) did not examine changes in PA behavior itself but rather in psychological or social variables associated with PA (e.g. motivational, intentional, or habitual variables); (iv) used PA as a moderator or mediator for another dependent variable (e.g. weight gain); or (v) dealt with daily hassles, non-events, or slow transitions. The study selection was an iterative process and criteria for inclusion and exclusion were further refined and adapted as knowledge on the topic grew, which is in line with the notion of a scoping review [28, 38].

### Data extraction and synthesis

Extracted data included the country in which the study was conducted, the life events and transitions covered, study design, sample characteristics, the employed PA assessment tools, and the PA domains and intensities that were examined (see S1 Table). If papers addressed several research questions, we focused on the data relevant to our research question. Moreover, if statistical models were adjusted (e.g. for sex, age, education, income, previous behavior, etc.), we report the results from these adjusted analyses assuming that this is a more *conservative*, and therefore, a more cautious approach of interpreting results. As our primary objective was to map the research landscape, we do not report on single mediation and interaction effects. As a first synthesis step, we provide a summary of the research landscape in which we also cluster the reported life events and transitions into distinct thematic categories. In a second step, the identified categories are used as a basis for a narrative synthesis of the key findings of the included studies (for both females and males if not otherwise specified). In order to stay true to the scoping nature of the present review and to delineate general trends, we refrained from summarizing results at the level of PA domains and intensities. Additionally, to be included in the narrative summary, results for events and transitions had to be reported in at least two studies. For the synthesis of the more general trends, results had to be presented in at least four studies.

## Results

**Fig 1:**
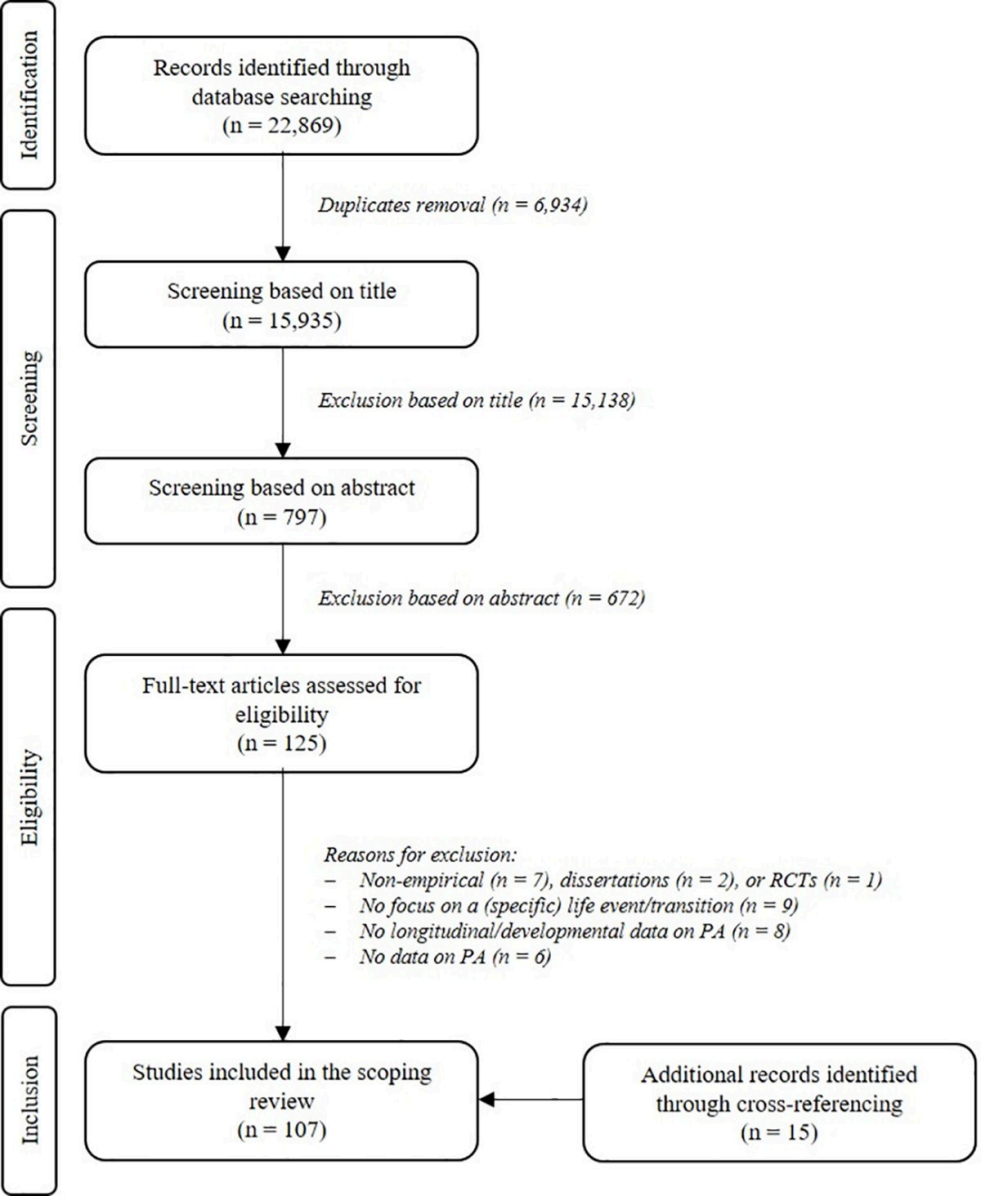
Flow chart of the systematic literature search. (adapted from the PRISMA flow diagram [39]).

The initial search yielded 22,869 articles with 15,935 remaining after duplicates were removed. After screening titles, abstracts, and full texts, 92 studies were included. Additionally, 15 records were identified through cross-referencing resulting in a total of 107 articles (Fig 1).

The included studies covered a total of 72 distinct life events and transitions, which we grouped into 10 thematic categories. For the life event *major personal achievement* [40], a clear allocation was not possible, since this event was considered to be rather broad and might occur within various categorical domains. Forty-nine life events or transitions (68%) were covered in only one or two studies. Table 3 summarizes, which life event categories, and which events and transitions in particular, were assessed across studies.

**Table 3:** Categories of life events and transitions covered across studies.

Category	Life Event/Transition	n (%)	Study (Citation No.)
Education-related events/transitions (n = 38; 35.5%)	Transition to primary school	5 (4.7%)	[41–45]
	Transition to secondary school	10 (9.3%)	[46–55]
	Transition from middle to high school	2 (1.9%)	[56, 57]
	Transition to post-secondary education/university/college	12 (11.2%)	[58–69]
	Return to study	3 (2.8%)	[40, 70, 71]
	(Post)graduation	8 (7.5%)	[59, 67, 70, 72–76]
	Receiving more education	1 (0.9%)	[77]
Employment-related events/transitions (n = 41; 38.3%)	Entry into labor market	10 (9.3%)	[40, 58, 59, 67, 70–72, 76, 78, 79]
	Changing jobs	6 (5.6%)	[40, 71, 72, 77, 80, 81]
	Quitting/stopping/losing job	7 (6.5%)	[40, 72, 77, 79, 80, 82, 83]
	Retirement	29 (27.1%)	[40, 80, 83–109]
	Increased/decreased income	2 (1.9%)	[40, 77]
	Going on welfare	1 (0.9%)	[72]
	Difficulty finding a job	1 (0.9%)	[40]
Health-related events/transitions (n = 19; 17.8%)	Developing/recovering from a major personal illness (e.g. cancer)/injury	10 (9.3%)	[40, 72, 80, 109–115]
	Developing/recovering from chronic diseases (e.g. overweight, diabetes, etc.)	5 (4.7%)	[77, 80, 115–117]
	Becoming normal weight	1 (0.9%)	[77]
	Menopausal transition	4 (3.7%)	[40, 118–120]
	Onset of menarche	1 (0.9%)	[121]
	Major surgery	2 (1.9%)	[40, 80]
	Involvement in a serious accident	2 (1.9%)	[40, 80]
	Developing/recovering from a depression	2 (1.9%)	[77, 80]
	Becoming (un)happy	1 (0.9%)	[77]
	Developing/recovering from a long-term-disability	2 (1.9%)	[77, 109]
	Developing/recovering from pain	2 (1.9%)	[77, 80]
	Starting/stopping to smoke	1 (0.9%)	[77]
	Starting/stopping to drink alcohol	1 (0.9%)	[77]
	Becoming (un)healthy	1 (0.9%)	[77]
	Becoming a caregiver	1 (0.9%)	[83]
Relationship-related events/transitions (n = 23; 21.5%)	Starting/stopping a relationship	5 (4.7%)	[40, 72, 76, 80, 122]
	Starting cohabitation	6 (5.6%)	[40, 70, 76, 78, 123, 124]
	Getting married	15 (14.0%)	[40, 59, 70–72, 76–78, 83, 109, 123–127]
	Getting divorced/separated	11 (10.3%)	[40, 71, 72, 80, 83, 122, 123, 125–128]
	Losing a spouse/partner/widowhood	9 (8.4%)	[40, 77, 80, 95, 123, 127–130]
	Re-marriage	3 (2.8%)	[127, 128, 130]
	Engagement	1 (0.9%)	[72]
	Infidelity of spouse/partner	1 (0.9%)	[40]
	Major decline in health of a spouse/partner	3 (2.8%)	[40, 114, 131]
	Spouse/partner quitting/stopping/losing job	1 (0.9%)	[40]
	Spouse’s/partner’s retirement	2 (1.9%)	[40, 132]
	Spouse/partner moving into an institution	1 (0.9%)	[40]
Family-related events/transitions (n = 28; 26.2%)	Pregnancy/birth of a first/subsequent child	24 (22.4%)	[40, 58, 59, 70–72, 76, 78, 79, 109, 124, 133–145]
	Becoming a single parent	2 (1.9%)	[40, 71]
	Miscarriage	1 (0.9%)	[40]
	Stillbirth	1 (0.9%)	[40]
	Having a child with a disability/serious illness	1 (0.9%)	[40]
	Death of a child	2 (1.9%)	[40, 83]
	Major conflict with teenage/older children	1 (0.9%)	[40]
	Increased hassles with parents	1 (0.9%)	[40]
	Parents getting divorced/separated/re-married	1 (0.9%)	[40]
	Serious conflict between family members	1 (0.9%)	[40]
	Major decline in health of close friends/family	1 (0.9%)	[40]
	Death of friend/family	5 (4.7%)	[40, 72, 80, 83, 109]
	Family member being arrested	1 (0.9%)	[40]
	Getting/losing social support	2 (1.9%)	[77, 115]
	Child/other family member leaving home	1 (0.9%)	[40]
	Birth of a grandchild	1 (0.9%)	[40]
Residence-related events/transitions (n = 10; 9.3%)	Moving	5 (4.7%)	[40, 72, 80, 83, 115]
	Moving to an institution	2 (1.9%)	[40, 146]
	Moving out of parents’ house/independence	6 (5.6%)	[40, 59, 67, 70, 72, 78]
	Moving back to parents’ house	1 (0.9%)	[70]
	Starting a mortgage	1 (0.9%)	[72]
Leisure-time related events/transitions (n = 1; 0.9%)	Starting/stopping a hobby	1 (0.9%)	[80]
	Starting/stopping to go on holiday trips	1 (0.9%)	[80]
Victimization-related events/transitions (n = 2; 1.9%)	Distressing harassment at work	1 (0.9%)	[40]
	Being pushed/grabbed/shoved/kicked/hit	1 (0.9%)	[40]
	Being forced to take part in an unwanted sexual activity	1 (0.9%)	[40]
	Being robbed	2 (1.9%)	[40, 80]
Criminal activity-related events/transitions (n = 2; 1.9%)	Being arrested/going to jail	1 (0.9%)	[72]
	Legal troubles/involvement in a court case	1 (0.9%)	[40]
Force majeure/material loss (n = 1; 0.9%)	Natural disaster	1 (0.9%)	[40]
	Major loss/damage of personal property	1 (0.9%)	[40]

Descriptive analyses of the publication landscape over the past twenty-two years revealed an increasing research interest in the impact of life events and transitions on PA with 74 studies being published in the nine years since the last review on this topic has been conducted [19]. More than half of the studies were conducted in the USA (n = 35; 32.7%), Canada (n = 13; 12.1%), and Australia (n = 12; 11.2%). Studies varied with regard to their design, with the vast majority being prospective longitudinal studies (n = 88; 82.2%), followed by retrospective assessments (n = 19; 17.8%). Follow-up time for the prospective longitudinal studies ranged from three months [146] to 25 years [83]. Sample sizes varied from n = 7 [98] to n = 81,925 [123]. The age of participants ranged from three [44] to 91 years [109, 132]. Detailed study characteristics are summarized in S1 Table. Studies using the same samples are summarized in S2 Table.

A great variety of PA assessment tools was used. Ninety studies (84.1%) employed questionnaires to determine changes in PA. In total, 31 distinct PA questionnaires were used across studies with the Godin Leisure-Time Exercise Questionnaire (n = 6) and the Active Australia Survey (n = 4; three of which used the same sample) being the most recurring tools. Thirty-four studies (31.8%) did not report, which questionnaires were employed. One study combined the questionnaire assessment with interviews. Twenty-three studies (21.5%) used accelerometers or pedometers to assess PA. Of these, seven studies combined questionnaires and accelerometers, eleven studies used only accelerometers (for four to ten consecutive days), and five studies used pedometers usually in combination with either an accelerometer or a questionnaire. As a result, changes in PA behavior were assessed in different ways either taking into account the amount of PA (e.g. frequency and duration, which was assessed either via accelerometers, an open question, or a Likert scale) or by simply asking whether participants engaged in PA or whether PA behavior has changed over time (e.g. yes/no).

**Fig 2:**
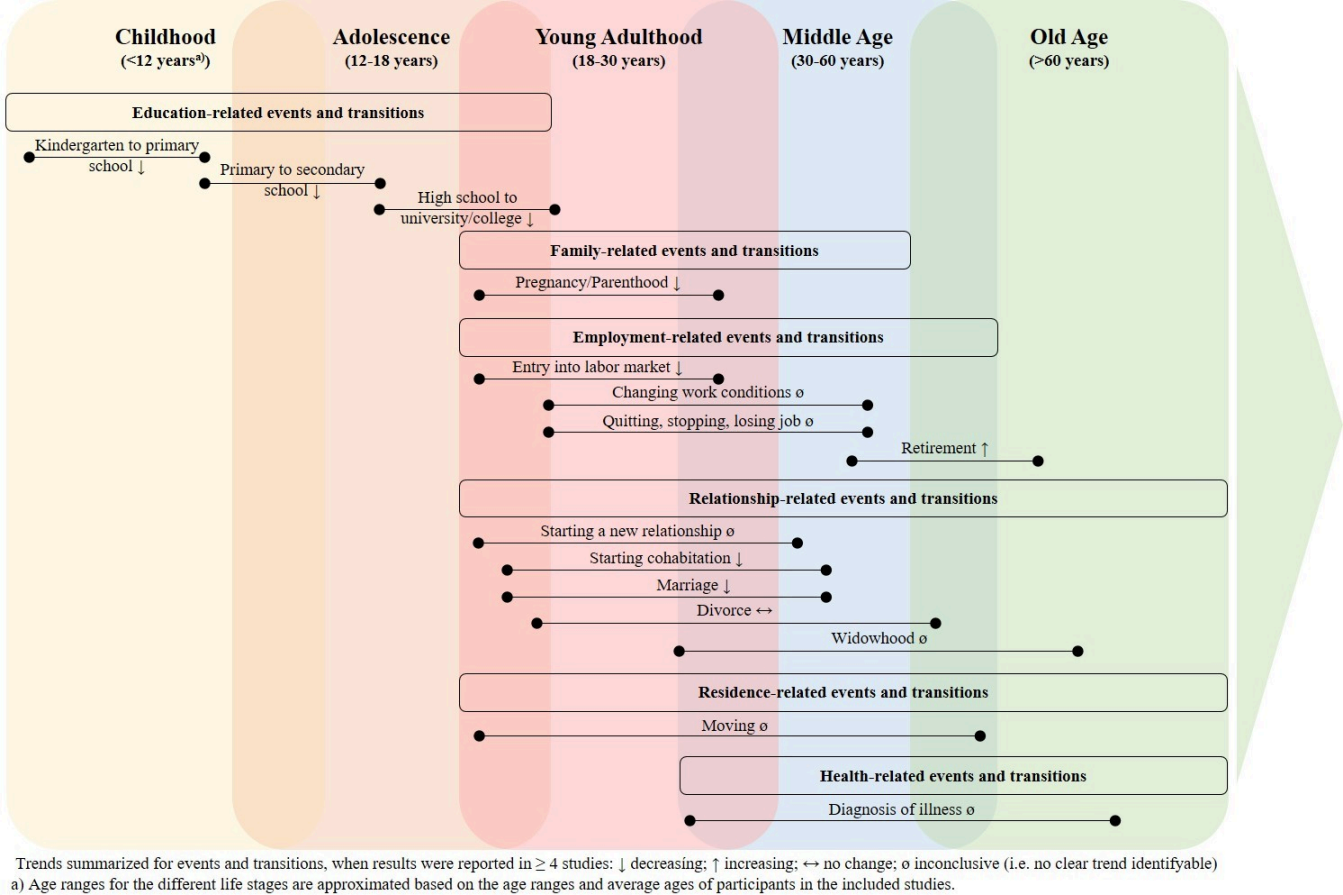
Summary of key findings of the impact of life events and transitions on PA across the life course.

Studies assessed seven more or less distinct PA domains: leisure-time PA (n = 80; 74.8%), occupational PA (n = 16; 15%), total PA (n = 22; 20.6%), walking and daily habitual PA (n = 41; 38.3%), commuting and active transport (n = 20; 18.7%), domestic activities (n = 20; 18.7%), and school activities (n = 8; 7.5%). While some studies focused on PA domains, others (additionally or instead) assessed PA intensities ranging from light (n = 18; 16.8%) to moderate (n = 39; 36.4%) to vigorous (n = 34; 31.8%) to moderate-to-vigorous PA (n = 29; 27.1%), and several combinations of the four intensity clusters (n = 38; 35.5%). As studies often merged several PA domains and intensities, a wide variety of combinations was apparent (for a detailed overview see S1 Table). In this regard, an in-depth and differentiated analysis of findings across all studies was not possible. Therefore, we abstracted from PA domains and intensities and summarized trends. The following sub-chapters focus on the associations between specific events and transitions and PA. Table 4 depicts, which of the included studies reported increases, decreases, or no changes in PA for a respective event or transition. We only summarized general trends for an event or transition when respective results were reported in at least four studies. As single studies assessed different PA domains and intensities, they might be reported multiple times (i.e. for increases, decreases, or no changes). Fig 2 summarizes trends for events and transitions across the life course.

**Table 4:** Trends and consistency of key findings across studies for events, when results were reported in ≥ 4 studies.

Life event/transition	Increase	Decrease	No change
***Education-related events/transitions***			
Kindergarten to primary school	**1** [42]	**5** [41–45]	**1** [41]
Primary to secondary school	**4** [46–48, 50]	**10** [47, 48, 50–57]	**4** [47–49, 51]
High school to university/college		**14** [58, 60–69, 73–75]	**2** [59, 65]
***Family-related events/transitions***			
Pregnancy, Parenthood^1^	**5** [79, 136, 142, 144, 145]	**20** [40, 59, 70, 71, 76, 78, 109, 124, 133, 134, 136–145]	**5** [124, 135, 139, 142, 144]
***Employment-related events/transitions***			
Entry into the labor market	**1** [72]	**7** [40, 58, 71, 72, 76, 78, 81]	**4** [59, 67, 77, 79]
Changing work conditions	**2** [40, 72]	**2** [71, 72]	
Quitting, stopping, losing job	**2** [40, 72]		**2** [79, 83]
Retirement	**25** [40, 84, 86–102, 104–109]	**11** [83–86, 93, 98, 103–107]	**8** [85–87, 92, 95, 96, 98, 103]
***Relationship-related events/transitions***			
Starting a new relationship	**2** [40, 72]	**3** [76, 122, 123]	**2** [72, 122]
Starting cohabitation		**3** [70, 76, 78]	**1** [124]
Marriage	**1** [125]	**9** [40, 59, 70–72, 76–78, 109]	**5** [72, 77, 83, 124, 126]
Divorce	**2** [126, 127]	**2** [40, 122]	**5** [71, 83, 122, 125, 128]
Widowhood	**3** [40, 129, 130]		**4** [77, 95, 127, 128]
***Health-related events/transitions***			
Diagnosis of illness^2^	**7** [77, 111–114, 117, 118]	**7** [40, 77, 109, 110, 109, 114, 117]	**2** [72, 114]
***Residence-related events/transitions***			
Moving	**1** [79]	**1** [109]	**2** [72, 83]

^1^Pregnancy and parenthood were subsumed as one event as several studies assessed PA changes across pregnancy including a postpartum period

^2^Diagnosis of illness also includes the diagnosis of a chronic condition

### Education-related events and transitions

Educational transitions were covered from early childhood (e.g. from kindergarten to school) to young adulthood (e.g. entry into post-secondary education or (post)graduation). Five studies assessed the transition from kindergarten to primary school and found mainly decreases in PA [41–45]. Two of these studies reported a rebound effect to or beyond pre-school levels [42, 44].

Similarly, findings on the transition from primary to secondary and from middle to high school showed mainly declines in PA across this transition [47, 48, 50–57]. Yet, studies also reported increases [46–48, 50] and no changes [47–49, 51] in PA, which was due to the assessment of different PA domains and intensities and whether activities occurred during weekdays or the weekend.

Studies, which focused on the transition from high school to post-secondary education (e.g. college or university) or leaving full-time education (e.g. started to work), showed that this is a period during which emerging adults are vulnerable to decreasing PA levels [58, 60, 61, 63–65, 67–69, 73, 76]. A greater proportion of high school graduates decreased their PA levels as opposed to those who increased PA [61, 62] and a shift from meeting PA guidelines to not meeting them occurred [75]. Two studies indicated that changes in PA were dependent on the environmental context, as high school graduates who started working were more likely to engage in moderate-to-vigorous and occupational PA than those who transitioned to post-secondary education although, in general, both groups tended to decrease their PA levels [73, 74].

### Employment-related events and transitions

Beginning paid work or entry into the labor market was mainly associated with decreases in PA. These declines were particularly pronounced in leisure-time PA [40, 58, 71, 72, 76, 78, 81], although two studies found contrasting findings for women and men [72, 77]. Four studies found no changes in PA [59, 67, 77, 79].

Changes in work conditions or jobs were associated with both decreases [71, 72] and increases [40, 72] in PA depending on domain, intensity, age, and gender. Similarly, losing or quitting a job or having a decreased income were associated with more [40, 72] and less [40] PA, while two studies reported no changes [79, 83]. These results again depended on PA domain, intensity, gender, and age. Being discharged from military was associated with a shift from 68.2% of participants meeting PA guidelines to 50.4% [82].

Retirement was the most examined life transition with 29 studies (27.1%). In general, retirement was associated with increases in leisure-time PA, (recreational) walking, and domestic activities [40, 84, 85, 87, 88, 90, 91, 93, 96, 99, 100, 103, 104, 106–109, 132] and decreases in occupational PA, active transportation, and total PA [83, 84, 93, 103, 104, 106, 107]. With regard to PA intensities, an increase especially in light and moderate PA occurred [87, 96], whereas findings on vigorous and moderate-to-vigorous PA were more ambiguous [93, 95, 96, 105, 132]. Qualitative data suggests that most retirees experience an increase in PA, while only a minority reports decreases or no changes at all [97] Prevalence data similarly indicates that there is an increased probability of meeting US government’s PA guidelines after retirement [94]. With regard to the type of retirement, findings were heterogeneous. Transitioning to full-time and voluntary retirement was linked to increased PA levels [89, 90], whereas transitioning to economic inactivity due to disability led to decreases [89, 105]. For the latter, however, one study reported declines in women only, whereas men increased their PA [96]. Retiring from a physically demanding job was associated with a decrease in PA [86], whereas for those retiring from a sedentary job, there was an increase [86]. Similarly, PA declined more in women and men from manual than for those from non-manual social classes [84]. Some studies reported on the long-term trajectories of PA patterns across the retirement transition indicating that PA tends to increase right after retirement but declines later on [91, 99, 102, 104, 105]. In contrast, two studies found evidence that PA levels remained stable after an initial increase [101, 108]. Findings for the pre-retirement period were ambiguous with reported increases and decreases in PA [102, 105] depending on domain and gender.

### Health-related events and transitions

Being diagnosed with a serious illness or disease was associated with mixed outcomes. Studies investigating changes in PA behavior after a cancer diagnosis reported both increases and decreases [110–113], while being diagnosed with a chronic disease or condition was linked to increases in PA [77, 117]. However, for older women, the diagnosis of an illness, having surgery, developing pain, and becoming obese or “(un)healthy” were associated with a decrease in PA [40, 77].

Additional health-related events and transitions included the menopausal transition, which was associated with decreases in PA in two studies [118, 119] and with no changes in one study [120], the onset of menarche, which was linked to a significant negative slope in PA (with the gynecological age-based model but not the chronological one) [121], and the onset of impaired sleep, which entailed an increased risk of becoming physically inactive [116]. These findings are in line with qualitative research suggesting that the onset of health and mobility problems is associated with constraints regarding PA [109].

### Relationship-related events and transitions

Findings on the impact of starting a new close relationship were mixed with two studies reporting increases in PA for young women [40] and men [72],two indicating decreases for both genders [76, 123], and one reporting decreases only for women [122] Ending a relationship was rather associated with increases in PA [72, 123] depending on domain and gender. Starting cohabitation was linked to decreases in PA [70, 76, 78], although one study found no changes [124]. Becoming engaged and getting married were mainly associated with decreases in PA [40, 59, 70–72, 76–78, 109, 126], depending on domain, intensity, and gender. Only one study found a positive relationship between marriage and PA outcomes [125]. Five studies reported no changes in PA, however, depending on domain, intensity, or gender [72, 77, 83, 124, 126]. Getting divorced or separated yielded mixed results, however, showing a trend towards no changes [71, 83, 122, 125, 128]. In addition, two studies reported decreases in PA in young women [40] and men [122], whereas two other studies indicated increases in PA [126, 127].

The death of a spouse and widowhood were associated with increases in PA in three studies [40, 129, 130] while four studies reported no changes [77, 95, 127, 128]. These relationships were dependent on PA domain, gender, and time since bereavement. Getting re-married and re-coupling were associated with both decreased [122, 128] and increased [130] levels of PA, again depending on domain and gender.

### Family-related events and transitions

Becoming a parent was primarily associated with decreases in PA [109, 124, 136], which were particularly pronounced in women [40, 70, 71, 139] and first-time parents [59, 76, 78, 124, 138, 142]. One study described the quality of change in PA as a smooth decline for women before and after birth, whereas men experienced a rather abrupt drop in the year after birth [140]. Expecting a second or subsequent child was associated with increases in light activities [142], yet, also with decreases in leisure-time PA [124]. Data on group trajectories reflects these tendencies as more women become inactive across the transition to parenthood than those who became active [133, 139]. Nevertheless, one retrospective study indicated that new parents might substitute activity domains, thus, not abandoning PA altogether and that parenthood might also present an opportunity to become more active [136].

Changes in PA were not only associated with parenthood but already with pregnancy as one study reported that substantially more women decreased their PA as opposed to those who increased theirs [137]. In general, pregnancy was linked to declines in PA [134, 141, 143–145]. Some studies, however, indicated increases in light activities such as walking or domestic chores [79, 141, 144, 145]. Results on long-term trajectories from pre-pregnancy to parenthood were ambiguous with some studies reporting rebound effects across the postpartum period [134, 141], one study reporting no changes in PA from pregnancy to postpartum [143], and one study indicating slight increases during maternity leave, which decreased again upon return-to-work [135]. Despite these trends, one study indicated that individual growth trajectories varied significantly from the average growth curve [134].

### Residence-related events and transitions

Moving out of the parental home and gaining residential independence was linked to decreases in PA [59, 78] or no changes [67]. Moving in general was associated with no changes [72, 83], but with increases in transport-related walking when moving to a less urbanized area [79]. Moving to an institution, such as retirement living, was similarly associated with significant decreases in PA [40, 146]. Qualitative data suggested that relocations were often perceived as disruptions in social networks, therefore leading to a drop in activities [109].

## Discussion

In the present scoping review, we examined the state of research on the impact of life events and transitions on PA behavior across the life course. In this regard, we mapped the research landscape and summarized key findings.

Life events and transitions that were covered by studies spanned from early childhood to old age and were thematically clustered into ten distinct categories. Childhood, adolescence, and young adulthood were life phases that displayed a wide variety of life events and transitions, that were oftentimes education-, relationship-, and family-related. For middle and older aged samples, the life events and transitions that were examined the most were illness, bereavement, and, above all, retirement.

Our results are mostly in line with previous reviews indicating that events and transitions during young adulthood are oftentimes accompanied by declines in PA [18–20, 23–26] and that retirement may offer a *window of opportunity* [105] for positive changes in PA despite declines in occupational PA and active commuting [19, 27]. Moreover, our results indicate a decreasing trend in PA for the transition from primary to secondary school. This corresponds to one review that reports results for six studies that we have included as well [22]. Additionally, despite including six more studies (including those for the transition from middle to high school), our results similarly indicate that changes in PA across this transition are dependent on PA domain, intensity, and time of the day or the week. Our review shows that for some life events and transitions (e.g. changing work conditions, quitting or losing a job, starting a new relationship, widowhood, moving, or diagnosis of illness), it is not possible to summarize clear trends. This issue has already been emphasized in the review by Engberg et al. [19] indicating that the causal and directional impact of life events and transitions on PA must not be overstated. If and how individuals adapt to the same event is a highly idiosyncratic process that depends on a multitude of factors (including psychosocial moderators and mediators that are oftentimes not assessed) and that might go hand in hand with increases, decreases, or no changes in PA at all. Moreover, life events and transitions might account for substitution effects regarding PA domains and intensities, thus, not rendering total PA.

### Limitations of current research and desiderates

Limitations in the current state of research can be summarized as theoretical shortcomings on the one hand and methodological issues on the other. Subsequently, we want to discuss these issues and suggest some potential future directions.

#### Theoretical limitations

On a theoretical and conceptual level, the majority of included studies neither precisely defined the terms *life event* or *transition* nor did they provide a theoretical framework that conceptually relates life events and PA behavior. The lack of terminological and theoretical framing became particularly apparent in vague terminology. For instance, some studies used the term life event for processes or *slow transitions* (such as difficulties finding a job, “becoming (un)happy”, or becoming overweight or obese), which would, according to our definition, not account for a life event as a change in status. Yet, these studies also assessed other events and were, therefore, included in this review. Similarly, some life events were rather unspecific and it was not clear what they entailed (e.g. “major personal achievement”, “receiving more education”, or “becoming (un)healthy”). Similar to the lack of conceptual clarity regarding life events, studies rarely reflected upon the concept of PA. Most studies acknowledged some sort of basic definition (mostly referring to Caspersen et al., [34]), however, they focused on a variety of different PA domains and intensities often resulting in inconclusive or mixed findings and making it rather difficult to synthesize results in a differentiated, yet concise fashion.

We think that the lack of conceptual clarity with regard to the terms *life event* and *transition* and the complexity of a phenomenon like PA need to be addressed in future research. Theoretical and terminological awareness are highly relevant as they help to guide research processes and embed empirical findings within a framework for interpretation that increases scientific rigor, reduces the risk of jumping to hasty conclusions, and allows for comparability across studies. Theories should therefore be at “the center of research” [147, p. 2] and play a key role in future research.

#### Methodological limitations

Terminological and conceptual disparities resulted in quite heterogeneous methodological approaches across studies as indicated by the variety of sampling procedures and life event assessment tools that were employed. Most studies chose participants, who found themselves in a period in which a given event or transition usually occurs (e.g. educational transitions or retirement). Others used demographic questionnaires to assess whether a life event (e.g. marriage, school enrollment, or change in employment status) had occurred in a given period. Finally, few studies used retrospective interviews or Life-Event-Scales that stress more on the subjective significance of an event. Independent of the methodological approach, the studies rarely provided theoretical justifications for the decision on how to assess a particular life event or transition.

Methodological heterogeneity also became evident with regard to the use of PA assessment tools. Thirty-one different PA questionnaires targeting various domains and intensities and using different question formats were applied. Additionally, 34 studies did not specify which scales were used. Objective PA assessment tools (e.g. accelerometers or pedometers) were used in only 21.5% of the studies–mostly with children and adolescents–and were rarely combined with self-reports on PA habits. This fragmented and inconsistent use of assessment tools and validated scales has already been indicated by Engberg et al. [19] and complicated the comparison of results across studies.

For future research, we consider it as highly important to be aware of the complexity of PA behavior and its distinct domains and intensities and to carefully choose respective assessment tools. Although a plethora of validated PA scales exists, almost 32% of the included studies did not specify, which questionnaires were used. Relying on validated scales that are able to differentiate between various modes of PA rather than on *on-the-fly* creations [148] may facilitate comparability across studies in the future. Moreover, it might be beneficial to combine objective and subjective PA assessment tools in order to measure PA levels, behavioral patterns, and subjective perceptions to fully understand how and why behavior changes.

The vast majority of studies were prospective longitudinal. These designs allow to identify patterns within populations and reveal relationships between life events and transitions and PA behavior. However, two issues might limit the informative value of prospective longitudinal studies. First, long periods between follow-up assessments pose a challenge to the identification of the exact timing of a life event meaning that immediate effects might remain hidden. Additionally, changes in PA levels could also be influenced by other circumstances and events that occurred within this period but were not assessed specifically. Second, if follow-up times are rather short, long-term developments and rebound or readjustment effects remain concealed.

To counteract these two potential limitations, prospective longitudinal studies should ideally use repeated measures over an extended follow-up time in order to capture life events and transitions in their temporal complexity and to account for both immediate and more persistent effects. Here, a temporal division of transition periods may be helpful. Studies on retirement, pregnancy, or menopause have shown that a distinction in a period before (pre), around (peri), and after an event (post) is beneficial in order to better understand the long-term trajectories of behavioral pathways. The temporal fragmentation in three phases may also help to assess the quality of behavioral changes across a transition, meaning whether a behavior changes in a smooth or rather abrupt fashion.

A further methodological limitation of current research is that most studies did not control for concomitant events but rather focused on singular events. Studies targeting young adulthood in particular have shown that this is a developmental period, which is prone to the co-occurrence or accumulation of various life events and transitions, which might all interact in changing PA behavior. A similar challenge that future research will have to deal with is to separate the effects of life events on PA behavior from underlying general trends. It has been widely acknowledged that PA declines with increasing age [149, 150] and that adolescence and young adulthood are periods where steep declines are to be expected [9]. In this regard, PA patterns might change due to biological maturation or other environmental factors that are not necessarily related to life events and transitions. Only few studies (mostly those with a focus on pregnancy, parenthood, and retirement) employed either long-term follow-ups or control groups to monitor these underlying trends. A careful choice of assessment tools, long-term follow-ups, control group designs, and more complex statistical models might be necessary to disentangle potential interaction effects between different life events and transitions, underlying trends, and other potential mediator or moderator effects (e.g. gender, age, socio-economic status, etc.).

In addition, we advocate that future research should further consider the use of biographical retrospective studies in order to assess life events and their impact on PA from an individualized, yet socio-cultural perspective [31]. It is highly relevant to add explanatory value to the rather descriptive results of prospective longitudinal studies. Biographical analyses may reveal how and why life events become critical events in the sense that they are valued as personally significant [31], how they are perceived and processed, how they account for changes in PA behavior across the life course, and which underlying meaning is attributed to these developments. While reconstructive and self-reported PA data might be limited due to potential recall bias or inaccuracy as well as social desirability [151, 152], the assessment of perceptions and attitudes about behavioral pathways adds another perspective to better understand when and why individuals take up or terminate PA.

With regard to sample characteristics, a total of 20 studies included only females while only two studies included solely men. As for the remaining studies, 17 showed an uneven gender distribution as more than 65% of the sample consisted of women. Our findings indicate that women and men may respond in different ways to certain events and transitions. However, as studies showed great diversity in methodological approaches and sampling, clear gender effects were difficult to identify.

Moreover, most studies examined samples of well-educated people making it difficult to generalize results for other populations. Particularly, research on retirement has shown that job characteristics may have an impact on the way PA behavior evolves across this transition. Therefore, future research should also focus on socioeconomic circumstances and apply a sampling strategy that also takes into account disadvantaged socioeconomic positions.

### Limitations of the review

With regard to our review, some limitations occur as well. First, the categorization of life events and transitions into ten thematic categories appeared to be the most reasonable, although we acknowledge that other categorical differentiations might be possible. Nevertheless, considering the heterogeneity of studies, we propose these initial distinctions in order to facilitate the synthesis of results. In this regard, however, a second limitation occurs. While the inclusion of 107 articles assessing 72 different life events and their relationship to various PA domains and intensities is a notable strength for mapping the research landscape on this topic, it also poses a considerable constraint for the depth of our analysis. Thus, the narrative synthesis of results does not reflect the entire complexity of the life event-PA relationship, but rather tries to identify emerging trends and draw out patterns across studies on a more general level. Third, only one author reviewed full-texts for eligibility. To include only studies that fit the inclusion criteria, arguable studies were discussed with a second researcher until consensus was reached. Fourth, we did not conduct a quality appraisal of the included studies. In this regard, studies were given the same weight when scoping the research landscape, although comparability might be limited. Fifth, although we extended the database search of two previous reviews [18, 19], studies that were published in another language or in journals that were not indexed in the databases might not have been found. Through cross-referencing we tried to lower the risk of missing any relevant studies. By focusing on peer-reviewed journal articles, potentially relevant grey literature (e.g. dissertations that are published as monographs) might have been missed. Eventually, by searching for terms such as *life event* or *transition*, studies focusing on certain events and transitions (e.g. parenthood, pregnancy, retirement, etc.) but not using the terms *life event* or *transition* might have been missed. Again, by cross-referencing, we aimed to lower this particular risk.

## Conclusion

This scoping review provides an overview of the current state of research on the impact of life events and transitions on PA and a summary of key findings in this area. Considering the health benefits of regular PA, we argue that it is crucial to understand when and why individuals take up or terminate PA. In this context, life events and transitions can be conceptualized as natural interventions that inevitably occur within the life course and that are oftentimes accompanied by changes in PA behavior for both the better and the worse. Despite some emerging trends, the overall state of research on this topic is still characterized by heterogeneity and fragmentation, which is reflected in a lack of theoretical, conceptual, and terminological clarity as well as in disparate study designs, methods, and reporting. This makes it difficult to synthesize and compare results across studies. Eventually, we hope that our synthesis of the research landscape will help to further develop research practices in the highly relevant fields of PA and sport, health, and leisure studies.

## Supporting information

S1 Table Study characteristics of the 107 included studies arranged by the assessed life event and transition categories. (DOCX)

S2 Table Studies assessing the same samples. (DOCX)

S1 File Preferred reporting items for systematic reviews and meta-analyses extension for scoping reviews (prisma-scr) checklist. (DOCX)

## References

[1] Warburton DER, Bredin SSD. Reflections on physical activity and health: What should we recommend? Can J Cardiol. 2016;32:495–504. 10.1016/j.cjca.2016.01.024 26995692

[2] Reiner M, Niermann C, Jekauc D, Woll A. Long-term health benefits of physical activity: A systematic review of longitudinal studies. BMC public health. 2013;13. 10.1186/1471-2458-13-813 PMC3847225 24010994

[3] Eime RM, Young JA, Harvey JT, Charity MJ, Payne WR. A systematic review of the psychological and social benefits of participation in sport for adults: Informing development of a conceptual model of health through sport. Int J Behav Nutr Phy. 2013;10(135). Epub 2013/12/10. 10.1186/1479-5868-10-135 24313992 PMC4028858

[4] Morgan AJ, Parker AG, Alvarez-Jimenez M, Jorm AF. Exercise and mental health: An exercise and sports science Australia commissioned review. J Exerc Physiol. 2013;16(4):64–73.

[5] Physical Activity Guidelines Advisory Committee. Physical Activity Guidelines Advisory Committee Scientific Report. Wahsington, DC: Physical Activity Guidelines Advisory Committee; 2018.

[6] Pettee Gabriel KK, Morrow JR, Woolsey A-L. Framework for physical activity as a complex and multidimensional behavior. J Phys Act Health. 2012;9(Suppl 1):S11–S8. 22287443 10.1123/jpah.9.s1.s11

[7] Hirvensalo M, Lintunen T. Life-course perspective for physical activity and sports participation. European Review of Aging and Physical Activity. 2011;8(1):13–22.

[8] European Commission. Special Eurobarometer 472: Sport and physical activity report. Survey requested by the European Commission, Directorate-General for Education, Youth, Sport and Culture and co-ordinated by the Directorate-General for Communication (DG COMM ´Media monitoring, Media Analysis and Eurobarometer´ Unit). Brussels: European Commission, Directorate-General for Education Y, Sport and Culture; 2018.

[9] Aaron DJ, Jekal Y-S, LaPorte RE. Epidemiology of physical activity from adolescence to young adulthood. In: Simopoulos AP, editor. Nutrition and fitness: Obesity, the metabolic syndrome, cardiovascular disease, and cancer. 94 Basel: Karger; 2005 p. 36–41. 10.1159/000088204 16145248

[10] Shaw BA, Liang J, Krause N, Gallant M, McGeever K. Age differences and social stratification in the long-term trajectories of leisure-time physical activity. J Gerontol Soc Sic. 2010;65B(6):756–66. 10.1093/geronb/gbq073 PMC2954334 20855534

[11] Rovio SP, Yang X, Kankaanpää A, Aalto V, Hirvensalo M, Telama R, et al Longitudinal physical activity trajectories from childhood to adulthodd and their determinants: The Young Finns Study. Scand J Med Sci Spor. 2018;28(3):1073–83. 10.1111/sms.12988 28981988

[12] Morseth B, Jorgensen L, Emaus N, Jacobsen BK, Wilsgaard T. Tracking of leisure time physical activity during 28 yr in adults: The Tromso Study. Med Sci Sport Exer. 2011;43(7):1229–34. 10.1249/MSS.0b013e3182084562 21131860

[13] Barnett TA, Gauvin L, Craig CL, Katzmaryk PT. Distinct trajectories of leisure time physical activity and predictors of trajectory class membership: A 22 year cohort study. Int J Behav Nutr Phy. 2008;5(57). 10.1186/1479-5868-5-57 PMC2613394 18990250

[14] Rauner A, Jekauc D, Mess F, Schmidt S, Woll A. Tracking physical activity in different settings from late childhood to early adulthood in Germany: The MoMo longitudinal study. BMC public health. 2015;15(391). 10.1186/s12889-015-1731-4 PMC4407713 25887314

[15] Shang B, Duan Y, Huang WY, Brehm W. Fluctuation: A common but neglected pattern of physical activity behaviour: An exploratory review of studies in recent 20 years. Eur J Sport Sci. 2018;18(2):266–78. 10.1080/17461391.2017.1417486 29334317

[16] Malina RM. Tracking of physical activity and physical fitness across the lifespan. Res Q Exercise Sport. 1996;67(3):48–57. 10.1080/02701367.1996.10608853 8902908

[17] Telama R. Tracking of physical activity from childhood to adulthood: A review. Obesity Facts. 2009;2(3):187–95. 10.1159/000222244 20054224 PMC6516203

[18] Allender S, Hutchinson L, Foster C. Life-change events and participation in physical activity: A systematic review. Health Promot Int. 2008;23(2):160–72. 10.1093/heapro/dan012 18364364

[19] Engberg E, Alen M, Kukkonen-Harjula K, Peltonen JE, Tikkanen HO, Pekkarinen H. Life events and change in leisure time physical activity: A systematic review. Sports Med. 2012;42(5):433–47. 10.2165/11597610-000000000-00000 22512413

[20] Condello G, Puggina A, Aleksovska K, Buck C, Burns C, Cardon G, et al Behavioral determinants of physical activity across the life course: A "DEterminants of DIet and Physical ACtivity" (DEDIPAC) umbrella systematic literature review. Int J Behav Nutr Phy. 2017;14(58). 10.1186/s12966-017-0510-2 28464958 PMC5414221

[21] Stults-Kolehmainen MA, Sinha R. The effects of stress on physical activity and exercise. Sports Med. 2014;44(1):81–121. 10.1007/s40279-013-0090-5 24030837 PMC3894304

[22] Chong KH, Parrish AM, Cliff DP, Kemp BJ, Zhang Z, Okely AD. Changes in physical activity, sedentary behaviour and sleep across the transition from primary to secondary school: A systematic review. J Sci Med Sport. 2019 10.1016/j.jsams.2019.12.002. 31848107

[23] Winpenny EM, Smith M, Penney T, Foubister C, Guagliano JM, Love R, et al Changes in physical activity, diet, and body weight across the education and employment transitions of early adulthood: A systematic review and meta-analysis. Obes Rev. 2020 10.1111/obr.12962. PMC7079102 31955496

[24] Bellows-Riecken KH, Rhodes RE. A birth of inactivity? A review of physical activity and parenthood. Prev Med. 2008;46:99–110. 10.1016/j.ypmed.2007.08.003 17919713

[25] Pot N, Renske K. Physical activity and sport participation: A systematic review of the impact of fatherhood. Preventive Medicine Reports. 2016;4:121–7. 10.1016/j.pmedr.2016.05.018 27413672 PMC4929128

[26] Corder K, Winpenny EM, Foubister C, Guagliano JM, Hartwig XM, Love R, et al Becoming a parent: A systematic review and meta-analysis of changes in BMI, diet, and physical activity. Obes Rev. 2020 10.1111/obr.12959. PMC7078970 31955517

[27] Barnett I, Van Sluijs E, Ogilvie D. Physical activity and transitioning to retirement: A systematic review. American journal of preventive medicine. 2012;43(3):329–36. 10.1016/j.amepre.2012.05.026 22898127 PMC3830178

[28] Arksey H, O'Malley L. Scoping studies: Towards a methodological framework. Int J Soc Res Method. 2005;8(1):19–32.

[29] Daudt HML, Van Mossel C, Scott SJ. Enhancing the scoping study methodology: A large, inter-professional team's experience with Arksey and O'Malley's framwork. BMC Med Res Methodol. 2013;13(48). 10.1186/1471-2288-13-48 PMC3614526 23522333

[30] Tricco A, Lillie E, Zarin W, O'Brien K, Colquhoun H, Levac D, et al PRISMA-Extension for Scoping Reviews (PRISMA-ScR): Checklist and explanation. Ann Intern Med. 2018;169(7):467–73. 10.7326/M18-0850 30178033

[31] Filipp S-H. Ein allgemeines Modell für die Analyse kritischer Lebensereignisse. In: Filipp S-H, editor. Kritische Lebensereignisse. 3rd ed Weinheim: Beltz; 1995 p. 3–52.

[32] Luhmann M, Hofmann W, Eid M, Lucas RE. Subjective well-being and adaptation to life events: A meta-analysis. J Pers Soc Psychol. 2012;102(3):592–615. 10.1037/a0025948 22059843 PMC3289759

[33] John JM, Gropper H, Thiel A. The role of critical life events in the talent development pathways of athletes and musicians: A systematic review. Psychol Sport Exerc. 2019;45 10.1016/j.psychsport.2019.101565.

[34] Caspersen CJ, Powell KE, Christenson GM. Physical activity, exercise, and physical fitness: Definitions and distinctions for health-related research. Public Health Rep. 1985;100(2):126–31. 3920711 PMC1424733

[35] PubMed. Life change events [MeSH subject heading scope note] 1977 [cited 2018 6 August]. Available from: https://www.ncbi.nlm.nih.gov/mesh/68008016.

[36] Schlossberg NK. A model of analyzing human adaptation to transition. Couns Psychol. 1981;9(2):2–18.

[37] Holmes TH, Rahe RH. The Social Readjustment Rating Scale. J Psychosom Res. 1967;11(2):213–8. 10.1016/0022-3999(67)90010-4 6059863

[38] Levac D, Colquhoun H, O'Brien K. Scoping studies: Advancing the methodology. Implement Sci. 2010;5(69). 10.1186/1748-5908-5-69 PMC2954944 20854677

[39] Moher D, Liberati A, Tetzlaff J, Altman DG, The PRISMA Group. Preferred reporting items for systematic reviews and meta-analyses: The PRISMA statement. PLoS Med. 2009;6(7). PMC3090117 21603045

[40] Brown WJ, Heesch KC, Miller YD. Life events and changing physical activity patterns in women at different life stages. Ann Behav Med. 2009;37(3):294–305. 10.1007/s12160-009-9099-2 2009-16969-005. 19506989

[41] Jáuregui A, Villalpando S, Rangel-Baltazar E, Castro-Hernández J, Lara-Zamudio Y, Méndez-Gómez-Humarán I. The physical activity level of Mexican children decreases upon entry to elementary school. Sauld Pública de México. 2011;53(3):228–36. 21829888

[42] Oja L, Jurimae T. Tracking of motor abilities, physical activity, and elementary motor skills during transition from preschool to school. Acta Kinesiologiae Universitatis Tartuensis. 2001;6:91–101. SPHS-803398.

[43] Sigmund E, Sigmundová D, El Ansari W. Changes in physical activity in pre-schoolers and first-grade children: Longitudinal study in the Czech Republic. Child: Care Health Dev. 2009;35(3):376–82. 10.1111/j.1365-2214.2009.00945.x 2009-05103-013. 19397600

[44] Taylor RW, Williams SM, Farmer VL, Taylor BJ. Changes in physical activity over time in young children: A longitudinal study using accelerometers. PLoS One. 2013;8(11):e81567 Epub 2013/11/28. 10.1371/journal.pone.0081567 24282607 PMC3839894

[45] Crane JR, Naylor P-J, Temple VA. The physical activity and sedentary behaviour patterns of children in kindergarten and grade 2. Children. 2018;5(10). 10.3390/children5100131 WOS:000448544400003. 30241367 PMC6210440

[46] Cooper AR, Jago R, Southward EF, Page AS. Active travel and physical activity across the school transition: The PEACH Project. Med Sci Sport Exer. 2012;44(10):1890–7. . 10.1249/MSS.0b013e31825a3a1e 22525779

[47] De Meester F, Van Dyck D, De Bourdeaudhuij I, Deforche B, Cardon G. Changes in physical activity during the transition from primary to secondary school in Belgian children: what is the role of the school environment? BMC public health. 2014;14:261 Epub 2014/03/22. 10.1186/1471-2458-14-261 24645802 PMC3995550

[48] D'Haese S, De Meester F, Cardon G, De Bourdeaudhuij I, Deforche B, Van Dyck D. Changes in the perceived neighborhood environment in relation to changes in physical activity: A longitudinal study from childhood into adolescence. Health Place. 2015;33:132–41. 10.1016/j.healthplace.2015.03.004 WOS:000353346600017. 25840351

[49] Garcia AW, Pender NJ, Antonakos CL, Ronis DL. Changes in physical activity beliefs and behaviors of boys and girls across the transition to junior high school. J Adolescent Health. 1998;22(5):394–402. 10.1016/S1054-139X(97)00259-0 1998-02380-005. 9589341

[50] Jago R, Page AS, Cooper AR. Friends and physical activity during the transition from primary to secondary school. Med Sci Sport Exer. 2012;44(1):111–7. . 10.1249/MSS.0b013e318229df6e 21697746

[51] Rutten C, Boen F, Seghers J. Changes in physical activity and sedentary behavior during the transition from elementary to secondary school. J Phys Act Health. 2014;11(8):1607–13. 10.1123/jpah.2012-0465 2015-07867-014. 24508853

[52] Pate RR, Dowda M, Dishman RK, Colabianchi N, Saunders RP, McIver KL. Change in children's physical activity: Predictors in the transition from elementary to middle school. American journal of preventive medicine. 2019;56(3):E65–E73. 10.1016/j.amepre.2018.10.012 WOS:000458728200001. 30655084 PMC6380938

[53] Ridley K, Dollman J. Changes in physical activity behaviour and psychosocial correlates unique to the transition from primary to secondary schooling in adolescent females: A longitudinal cohort study. Int J Env Res Pub He. 2019;16(24):4959 10.3390/ijerph16244959 . 31817663 PMC6950115

[54] Marks J, Barnett LM, Strugnell C, Allender S. Changing from primary to secondary school highlights opportunities for school environment interventions aiming to increase phsyical activity and reduce sedentary behaviour: A longitudinal cohort study. Int J Behav Nutr Phy. 2015;12(59). 10.1186/s12966-015-0218-0 25952318 PMC4436807

[55] Barr-Anderson DJ, Flynn JI, Dowda M, Taverno Ross SE, Schenkelberg MA, Reid LA, et al The modifying effects of race/ethnicity and socioeconomic status on the change in physical activity from elementary to middle school. J Adolescebt Health. 2017;61:562–70. 10.1016/j.jadohealth.2017.05.007 PMC5654669 28732715

[56] Taymoori P, Berry TR, Lubans DR. Tracking of physical activity during middle school transition in Iranian adolescents. Health Educ J. 2012;71(6):631–41. .

[57] Shull ER, Dowda M, Saunders RP, McIver K, Pate RR. Sport participation, physical activity and sedentary behavior in the transition from middle school to high school. J Sci Med Sport. 2019:S1440-2440(19)30586-9. 10.1016/j.jsams.2019.10.017 . 31722841 PMC7054172

[58] Larouche R, Laurencelle L, Shephard RJ, Trudeau F. Life transitions in the waning of physical activity from childhood to adult life in the Trois-Rivières study. J Phys Act Health. 2012;9(4):516–24. 2012-12401-003. 10.1123/jpah.9.4.516 22592870

[59] Miller J, Nelson T, Barr-Anderson DJ, Christop MJ, Winkler M, Neumark-Sztainer D. Life events and longitudinal effects on physical activity: Adolescence to adulthood [published ahead of print]. Med Sci Sport Exer. 2018. 10.1249/MSS.0000000000001839 PMC7166312 30673690

[60] Bray SR. Self-efficacy for coping with barriers helps students stay physically active during transition to their first year at a university. Res Q Exercise Sport. 2007;78(2):61–70. 10.1080/02701367.2007.10599404 WOS:000245878900009. 17479575

[61] Bray SR, Born HA. Transition to university and vigorous physical activity: Implications for health and psychological well-being. J Am Coll Health. 2004;52(4):181–8. 10.3200/JACH.52.4.181-188 . 15018429

[62] Diehl K, Hilger J. Physical activity and the transition from school to university: A cross-sectional survey among university students in Germany. Sci Sport. 2016;31(4):223–6. .

[63] Van Dyck D, Bourdeaudhuij I, Deliens T, Deforche B. Can changes in psychosocial factors and residency explain the decrease in physical activity during the transition from high school to college or university? Int J Behav Med. 2015;22(2):178–86. 10.1007/s12529-014-9424-4 . 25031186

[64] Han JL, Dinger MK, Hull HR, Randall NB, Heesch KC, Fields DA. Changes in women's physical activity during the transition to college. Am J Health Educ. 2008;39(4):194–9. .

[65] Pullman AW, Masters RC, Zalot LC, Carde LE, Saraiva MM, Dam YY, et al Effect of the transition from high school to university on anthropometric and lifestyle variables in males. Appl Physiol Nutr Med. 2009;34(2):162–71. 10.1139/h09-007 WOS:000265604700009. 19370046

[66] Ullrich-French S, Cox AE, Bumpus MF. Physical activity motivation and behavior across the transition to university. Sport Exerc Perform Psychol. 2013;2(2):90–101. 10.1037/a0030632 2012-31767-001.

[67] Simons D, Rosenberg M, Salmon J, Knuiman M, Granich J, Deforche B, et al Psychosocial moderators of associations between life events and changes in physical activity after leaving high school. Prev Med. 2015;72:30–3. 10.1016/j.ypmed.2014.12.039 2015-10076-007. 25575797

[68] Parra-Saldias M, Castro-Pinero J, Castillo Paredes A, Palma Leal X, Diaz Martinez X, Rodriguez-Rodriguez F. Active commuting behaviours from high school to university in Chile: A retrospective study. Int J Env Res Pub He. 2019;16(1). 10.3390/ijerph16010053 WOS:000459111400053. 30587802 PMC6338952

[69] Deforche B, Van Dyck D, Deliens R, De Bourdeaudhuij I. Changes in weight, physical activity, sedentary behaviour and dietary intake during the transition to higher education: A prospective study. Int J Behav Nutr Phy. 2015;12(16). 10.1186/s12966-015-0173-9. PMC4332914 25881147

[70] Bell S, Lee C. Emerging adulthood and patterns of physical activity among young Australian women. Int J Behav Med. 2005;12(4):227–35. 10.1207/s15327558ijbm1204_3 2005-14018-003. 16262541

[71] Brown WJ, Trost SG. Life transitions and changing physical activity patterns in young women. American journal of preventive medicine. 2003;25(2):140–3. 10.1016/s0749-3797(03)00119-3 WOS:000184296200010. 12880882

[72] Paluch AE, Shook RP, Hand GA, O'Connor DP, Wilcox S, Drenowatz C, et al The influence of life events and psychological stress on objectively measured physical activity: A 12-month longitudinal study. J Phys Act Health. 2018;15(5):374–82. 10.1123/jpah.2017-0304 . 29485924

[73] Li K, Liu D, Haynie D, Gee B, Chaurasia A, Seo DC, et al Individual, social, and environmental influences on the transitions in physical activity among emerging adults. BMC public health. 2016;16:682 Epub 2016/08/04. 10.1186/s12889-016-3368-3 27485724 PMC4970300

[74] Molina-García J, Queralt A, Castillo I, Sallis JF. Changes in physical activity domains during the transition out of high school: Psychosocial and environmental correlates. J Phys Act Health. 2015;12(10):1414–20. 10.1123/jpah.2014-0412 2016-56936-004. 25599110

[75] Owens CS, Crone D, De Ste Croix MBA, Gidlow CJ, James DVB. Physical activity and screen time in adolescents transitioning out of compulsory education: a prospective longitudinal study. J Public Health. 2014;36(4):599–607. 10.1093/pubmed/fdt123 WOS:000345840900013. 24365762

[76] Van Houten JMA, Kraaykamp G, Pelzer BJ. The transition to adulthood: a game changer!? A longitudinal analysis of the impact of five major life events on sport participation. European Journal for Sport and Society. 2019;16(1):44–63. 10.1080/16138171.2019.1603832 WOS:000471956800004.

[77] Dai SL, Wang F, Morrison H. Predictors of decreased physical activity level over time among adults A longitudinal study. American journal of preventive medicine. 2014;47(2):123–30. 10.1016/j.amepre.2014.04.003 WOS:000339687300003. 24877993

[78] Van Houten JMA, Kraaykamp G, Breedveld K. When do young adults stop practising a sport? An event history analysis on the impact of four major life events. Int Rev Sociol Sport. 2017;52(7):858–74. .

[79] Gao J, Kamphuis CBM, Ettema D, Helbich M. Longitudinal changes in transport-related and recreational walking: The role of life events. Transport Res D-Tr E. 2019;77:243–51. 10.1016/j.trd.2019.11.006 WOS:000503099300018.

[80] Kenter EJ, Gebhardt WA, Lottman I, van Rossum M, Bekedam M, Crone MR. The influence of life events on physical activity patterns of Dutch older adults: A life history method. Psychol Health. 2014;30(6):627–51. 10.1080/08870446.2014.934687 2015-17462-002. 24942133

[81] Kirk MA, Rhodes RE. Physical activity status of academic professors during their early career transition: An application of the theory of planned behavior. Psychol Health Med. 2012;17(5):551–64. 10.1080/13548506.2011.647700 2012-21298-005. 22348598

[82] Littman AJ, Jacobson IG, Boyko EJ, Smith TC. Changes in meeting physical activity guidelines after discharge from the military. J Phys Act Health. 2015;12(5):666–74. 10.1123/jpah.2013-0260 2015-36453-011. 24828972

[83] Richards EA, Thomas PA, Forster AK, Hass Z. A longitudinal examination of the impact of major life events on physical activity. Health Educ Behav. 2019;46(3):398–405. 10.1177/1090198118822712 WOS:000470856600003. 30630375

[84] Barnett I, van Sluijs E, Ogilvie D, Wareham NJ. Changes in household, transport and recreational physical activity and television viewing time across the transition to retirement: longitudinal evidence from the EPIC-Norfolk cohort. J Epidemiol Commun H. 2014;68(8):747–53. 10.1136/jech-2013-203225 WOS:000339723200010. 24302753 PMC4112431

[85] Berger U, Der G, Mutrie N, Hannah MK. The impact of retirement on physical activity. Ageing Soc. 2005;25(2):181–95.

[86] Chung S, Domino ME, Stearns SC, Popkin BM. Retirement and physical activity: Analyses by occupation and wealth. American journal of preventive medicine. 2009;36(5):422–8. 10.1016/j.amepre.2009.01.026 19269129

[87] Ding D, Grunseit AC, Chau JY, Vo K, Byles J, Bauman AE. Retirement: A transition to a healthier lifestyle?: Evidence from a large Australian study. American journal of preventive medicine. 2016;51(2):170–8. 10.1016/j.amepre.2016.01.019 2016-36234-006. 26972491

[88] Evenson KR, Rosamond WD, Cai J, Diez-Roux AV, Brancati FL. Influence of retirement on leisure-time phyiscal activity: The Atherosclerosis Risk in Communities Study. Am J Epidemiol. 2002;155(8):692–9. 10.1093/aje/155.8.692 11943686

[89] Feng XQ, Croteau K, Kolt GS, Astell-Burt T. Does retirement mean more physical activity? A longitudinal study. BMC public health. 2016;16 10.1186/s12889-016-3253-0 WOS:000379963200002. 27439914 PMC4955250

[90] Henkens K, van Solinge H, Gallo WT. Effects of retirement voluntariness on changes in smoking, drinking and physical activity among Dutch older workers. Eur J Public Health. 2008;18(6):644–9. 10.1093/eurpub/ckn095 2008-17028-021. 18927184 PMC2727140

[91] Holstila A, Manty M, Rahkonen O, Lahelma E, Lahti J. Statutory retirement and changes in self-reported leisure-time physical activity: a follow-up study with three time-points. BMC public health. 2017;17(1):528 Epub 2017/06/01. 10.1186/s12889-017-4455-9 28558730 PMC5450199

[92] Jones SA, Li Q, Aiello AE, O'Rand AM, Evenson KR. Correlates of changes in walking during the retirement transition: The Multi-Ethnic Study of Atherosclerosis. Prev Med Rep. 2018a;11:221–30. 30210994 10.1016/j.pmedr.2018.07.002 PMC6129965

[93] Jones SA, Li QF, Aiello AE, O'Rand AM, Evenson KR. Physical activity, sedentary behavior, and retirement: The Multi-Ethnic Study of Atherosclerosis. American journal of preventive medicine. 2018b;54(6):786–94. 10.1016/j.amepre.2018.02.022 WOS:000432474500007. 29650285 PMC5962425

[94] Kämpfen F, Maurer J. Time to burn (calories)? The impact of retirement on physical activity among mature Americans. J Health Econ. 2016;45:91–102. 10.1016/j.jhealeco.2015.12.001 26773282

[95] Koeneman MA, Chinapaw MJM, Verheijden MW, van Tilburg TG, Visser M, Deeg DJH, et al Do major life events influence physical activity among older adults: The Longitudinal Aging Study Amsterdam. Int J Behav Nutr Phy. 2012;9 10.1186/1479-5868-9-147 2013-04232-001. 23245568 PMC3542084

[96] Lahti J, Laaksonen M, Lahelma E, Rahkonen O. Changes in leisure-time physical activity after transition to retirement: A follow-up study. Int J Behav Nutr Phy. 2011;8. 2012-32524-001. 10.1186/1479-5868-8-36 PMC3094268 21513555

[97] McDonald S, O'Brien N, White M, Sniehotta FF. Changes in physical activity during the retirement transition: A theory-based, qualitative interview study. Int J Behav Nutr Phy. 2015;12. 2015-09403-001. 10.1186/s12966-015-0186-4 PMC4343052 25889481

[98] McDonald S, Vieira R, Godfrey A, O'Brien N, White M, Sniehotta FF. Changes in physical activity during the retirement transition: A series of novel n-of-1 natural experiments. Int J Behav Nutr Phy. 2017;14 10.1186/s12966-017-0623-7 2017-55693-001. 29221449 PMC5723062

[99] Menai M, Fezeu L, Charreire H, Kesse-Guyot E, Touvier M, Simon C, et al Changes in sedentary behaviours and associations with physical activity through retirement: A 6-year longitudinal study. PLoS One. 2014;9(9). 10.1371/journal.pone.0106850 PMC4178017 25259801

[100] Oshio T, Kan M. The dynamic impact of retirement on health: Evidence from a nationwide ten-year panel survey in Japan. Prev Med. 2017;100:287–93. 10.1016/j.ypmed.2017.04.007 2017-25313-043. 28583660

[101] Schönbach JK, Pfinder M, Bornhorst C, Zeeb H, Brand T. Changes in sports participation across transition to retirement: Modification by migration background and acculturation status. Int J Env Res Pub He. 2017;14(11). 10.3390/ijerph14111356 WOS:000416545200072. 29117151 PMC5707995

[102] Sjösten N, Kivimaki M, Singh-Manoux A, Ferrie JE, Goldberg M, Zins M, et al Change in physical activity and weight in relation to retirement: the French GAZEL Cohort Study. BMJ open. 2012;2:e000522 Epub 2012/02/10. 10.1136/bmjopen-2011-000522 22318663 PMC3277904

[103] Slingerland AS, van Lenthe FJ, Jukema JW, Kamphuis CBM, Looman C, Giskes K, et al Aging, retirement, and changes in physical activity: Prospective cohort findings from the GLOBE study. Am J Epidemiol. 2007;165(12):1356–63. 10.1093/aje/kwm053 WOS:000247240700004. 17420180

[104] Sprod J, Olds T, Brown W, Burton N, van Uffelen J, Ferrar K, et al Changes in use of time across retirement: A longitudinal study. Maturitas. 2017;100:70–6. 10.1016/j.maturitas.2017.02.018 WOS:000406731800009. 28539179

[105] Stenholm S, Pulakka A, Kawachi I, Oksanen T, Halonen JI, Aalto V, et al Changes in physical activity during transition to retirement: a cohort study. Int J Behav Nutr Phy. 2016;13 10.1186/s12966-016-0375-9 WOS:000374142400001. 27084334 PMC4833915

[106] Touvier M, Bertrais S, Charreire H, Vergnaud A-C, Hercberg S, Oppert J-M. Changes in leisure-time physical activity and sedentary behaviour at retirement: A prospective study in middle-aged French subjects. Int J Behav Nutr Phy. 2010;7 10.1186/1479-5868-7-14 2012-31867-001. 20181088 PMC2834610

[107] Van Dyck D, Cardon G, De Bourdeaudhuij I. Longitudinal changes in physical activity and sedentary time in adults around retirement age: what is the moderating role of retirement status, gender and educational level? BMC public health. 2016;16(1):1125 Epub 2016/10/30. 10.1186/s12889-016-3792-4 PubMed Central PMCID: PMC5084354. 27793134 PMC5084354

[108] Henning G, Stenling A, Bielak AAM, Bjälkebring P, Gow AJ, Kivi M, et al Towards an active and happy retirement? Changes in leisure activity and depressive symptoms during the retirement transition. Aging Ment Health. 2020:1–11. 10.1080/13607863.2019.1709156 . 31965817

[109] Colley K, Currie MJB, Irvine KN. Then and now: Examining older people's engagement in outdoor recreation across the life course. Leisure Sci. 2019;41(3):186–202. 10.1080/01490400.2017.1349696 WOS:000472067400004.

[110] Blanchard CM, Denniston MM, Baker F, Ainsworth SR, Courneya KS, Hann DM, et al Do adults change their lifestyle behaviors after a cancer diagnosis? Am J Health Behav. 2003;27(3):245–56. 10.5993/ajhb.27.3.6 12751621

[111] Cerimagic S, Ahmadi N, Gurney H, Hossack T, Patel MI. Positive lifestyle changes following urological cancer diagnoses: An Australian interview based study. International Journal of Human Rights in Health Care. 2015;8(2):110–9. 10.1108/ijhrh-10-2014-0027 WOS:000218448000006.

[112] Humpel N, Magee C, Jones SC. The impact of a cancer diagnosis on the health behaviors of cancer survivors and their family and friends. Supportive care in cancer: official journal of the Multinational Association of Supportive Care in Cancer. 2007;15(6):621–30. Epub 2007/01/06. 10.1007/s00520-006-0207-6 . 17205274

[113] Satia JA, Campbell MK, Galanko JA, James A, Carr C, Sandler RS. Longitudinal changes in lifestyle behaviors and health status in colon cancer survivors. Cancer epidemiology, biomarkers & prevention: a publication of the American Association for Cancer Research, cosponsored by the American Society of Preventive Oncology. 2004;13(6):1022–31. Epub 2004/06/09. . 15184259

[114] Li KK, Cardinal BJ, Acock AC. Concordance of physical activity trajectories among middle-aged and older married couples: Impact of diseases and functional difficulties. J Gerontol B-Psychol. 2013;68(5):794–806. 10.1093/geronb/gbt068 WOS:000323440100015. 23873967

[115] Kostamo K, Vesala KM, Hankonen N. What triggers changes in adolescents' physical activity? Analysis of critical incidents during childhood and youth in student writings. Psychol Sport Exerc. 2019;45 10.1016/j.psychsport.2019.101564 WOS:000491218300025.

[116] Clark AJ, Salo P, Lange T, Jennum P, Virtanen M, Pentti J, et al Onset of impaired sleep as a predictor of change in health-related behaviours; analysing observational data as a series of non-randomized pseudo-trials. Int J Epidemiol. 2015;44(3):1027–37. Epub 2015/05/15. 10.1093/ije/dyv063 . 25969504

[117] Zhou PL, Hughes AK, Grady SC, Fang L. Physical activity and chronic diseases among older people in a mid-size city in China: A longitudinal investigation of bipolar effects. BMC public health. 2018;18 10.1186/s12889-018-5408-7 WOS:000429856400002. 29650011 PMC5898068

[118] Duval K, Prud'homme D, Rabasa-Lhoret R, Strychar I, Brochu M, Lavoie JM, et al Effects of the menopausal transition on energy expenditure: A MONET Group Study. Eur J Clin Nutr. 2013;67(4):407–11. 10.1038/ejcn.2013.33 WOS:000317036600018. 23422924 PMC4977179

[119] Lovejoy JC, Champagne CM, de Jonge L, Smith SR. Increased visceral fat and decreased energy expenditure during the menopausal transition. Int J Obesity. 2008;32:949–58. 10.1038/ijo.2008.25 PMC2748330 18332882

[120] Moilanen JM, Aalto AM, Raitanen J, Hemminki E, Aro AR, Luoto R. Physical activity and change in quality of life during menopause: An 8-year follow-up study. Health Qual Life Out. 2012;10 10.1186/1477-7525-10-8 WOS:000302066200001. 22269072 PMC3311608

[121] Beal SJ, Grimm KJ, Dorn LD, Susman EJ. Morningness–eveningness and physical activity in adolescent girls: Menarche as a transition point. Child Dev. 2016;87(4):1106–14. 10.1111/cdev.12539 2016-34140-014. 27097124 PMC4939120

[122] Salin K, Hirvensalo M, Kankaanpaa A, Magnussen CG, Yang X, Hutri-Kahonen N, et al Associations of partnering transition and socioeconomic status with a four-year change in daily steps among Finnish adults. Scand J Public Healt. 2019;47(7):722–9. 10.1177/1403494818807558 WOS:000503081200006. 30328367

[123] Josefsson K, Elovainio M, Stenholm S, Kawachi I, Kauppi M, Aalto V, et al Relationship transitions and change in health behavior: A four-phase, twelve-year longitudinal study. Soc Sci Med. 2018;209:152–9. 10.1016/j.socscimed.2018.03.006 WOS:000438481700018. 29566960

[124] Hull EE, Rofey DL, Robertson RJ, Nagle EF, Otto AD, Aaron DJ. Influence of marriage and parenthood on physical activity: A 2-year prospective analysis. J Phys Act Health. 2010;7(5):577–83. 2011-00092-002. 10.1123/jpah.7.5.577 20864752 PMC3124092

[125] King AC, Kiernan M, Ahn DK, Wilcox S. The effects of marital transitions on changes in physical activity: Results from a 10-year community study. Ann Behav Med. 1998;20(2):64–9. 10.1007/BF02884450 WOS:000078467900003. 9989310

[126] Kutob RM, Yuan NP, Wertheim BC, Sbarra DA, Loucks EB, Nassir R, et al Relationship between marital transitions, health behaviors, and health indicators of postmenopausal women: Results from the Women's Health Initiative. J Womens Health. 2017;26(4):313–20. 10.1089/jwh.2016.5925 2017-17299-006. 28072926 PMC5397241

[127] Lee S, Cho E, Grodstein F, Kawachi I, Hu FB, Colditz GA. Effects of marital transitions on changes in dietary and other health behaviours in US women. Int J Epidemiol. 2005;34(1):69–78. WOS:000227129600016. 10.1093/ije/dyh258 15231759

[128] Eng PM, Kawachi I, Fitzmaurice G, Rimm EB. Effects of marital transitions on changes in dietary and other health behaviours in US male health professionals. J Epidemiol Commun H. 2005;59(1):56–62. 10.1136/jech.2004.020073 2004-22057-005. 15598728 PMC1763358

[129] Stahl ST, Schulz R. The effect of widowhood on husbands’ and wives’ physical activity: The cardiovascular health study. J Behav Med. 2014;37(4):806–17. 10.1007/s10865-013-9532-7 2013-30780-001. 23975417 PMC3932151

[130] Wilcox S, Evenson KR, Aragaki A, Wassertheil-Smoller S, Mouton CP, Loevinger BL. The effects of widowhood on physical and mental health, health behaviors, and health outcomes: The Women's Health Initiative. Health Psychol. 2003;22(5):513–22. 10.1037/0278-6133.22.5.513 2003-08468-011. 14570535

[131] Ezendam NPM, Karlsen RV, Christensen J, Tjonneland A, van de Poll-Franse LV, von Heymann-Horan A, et al Do people improve health behavior after their partner is diagnosed with cancer? A prospective study in the Danish diet, Cancer and Health Cohort. Acta Oncol. 2019;58(5):700–7. 10.1080/0284186X.2018.1557342 WOS:000469362200022. 30706752

[132] Mueller T, Shaikh M. Your retirement and my health behavior: Evidence on retirement externalities from a fuzzy regression discontinuity design. J Health Econ. 2018;57:45–59. 10.1016/j.jhealeco.2017.10.005 WOS:000430775600004. 29182934

[133] Albright CL, Maddock JE, Nigg CR. Physical activity before pregnancy and following childbirth in a multiethnic sample of healthy women in Hawaii. Women Health. 2005;42(3):95–110. 10.1300/j013v42n03_06 16901890

[134] Cramp AG, Bray SR. Pre- and postnatal women's leisure time physical activity patterns. Res Q Exercis Sport. 2009;80(3):403–11. 10.1080/02701367.2009.10599578 19791626

[135] Grace SL, Williams A, Stewart DE, Franche RL. Health-promoting behaviors through pregnancy, maternity leave, and return to work: Effects of role spillover and other correlates. Women Health. 2006;43(2):51–72. 10.1300/J013v43n02_04 WOS:000242260000004. 17000611

[136] Hamilton K, White KM. Understanding parental physical activity: Meanings, habits, and social role influence. Psychol Sport Exerc. 2010;11(4):275–85. 10.1016/j.psychsport.2010.02.006 WOS:000279338500003.

[137] Hinton PS, Olson CM. Predictors of pregnancy-associated change in physical activity in a rural white population. Matern Child Heal J. 2001;5(1):7–14. 10.1023/a:1011315616694 11341722

[138] Hull EE, Garcia JM, Kolen AM, Robertson RJ. Parenthood and physical activity in young adults: A qualitative study. J Phys Act Health. 2015;12(6):782–8. 2015-44378-007. 10.1123/jpah.2013-0412 25133320

[139] McIntyre CA, Rhodes RE. Correlates of leisure-time physical activity during transitions to motherhood. Women Health. 2009;49(1):66–83. 10.1080/03630240802690853 2009-03304-005. 19485235

[140] Perales F, del Pozo-Cruz J, del Pozo-Cruz B. Long-term dynamics in physical activity behaviour across the transition to parenthood. Int J Public Health. 2015;60(3):301–8. 10.1007/s00038-015-0653-3 2015-02935-001. 25603985

[141] Pereira MA, Rifas-Shiman SL, Kleinman KP, Rcih-Edwards JW, Peterson KE, Gillman MW. Predictors of change in physical activity during and after pregnancy. American journal of preventive medicine. 2007;32(4):312–9. 10.1016/j.amepre.2006.12.017 17383562 PMC1894953

[142] Rhodes RE, Blanchard CM, Benoit C, Levy-Milne R, Naylor PJ, Downs D, et al Physical activity and sedentary behavior across 12 months in cohort samples of couples without children, expecting their first child, and expecting their second child. J Behav Med. 2014;37(3):533–42. 10.1007/s10865-013-9508-7 WOS:000336333300017. 23606310

[143] Symons Downs D, Hausenblas HA. Women's exercise beliefs and behaviors during their pregnancy and postapartum. J Midwifery Womens Health. 2004;49(2):138–44. 10.1016/j.jmwh.2003.11.009 15010667

[144] Treuth MS, Butte NF, Puyau M. Pregnancy-related changes in physical activity, fitness, and strength. Med Sci Sport Exer. 2005;37(5):832–7. 10.1249/01.mss.0000161749.38453.02 15870638

[145] Sjögren Forss K, Stjernberg L. Physical activity patterns among women and men during pregnancy and 8 months postpartum compared to pre-pregnancy: A longitudinal study. Front Public Health. 2019;7:294-. 10.3389/fpubh.2019.00294 . 31750283 PMC6843064

[146] Regan K, Intzandt B, Swatridge K, Myers A, Roy E, Middleton LE. Changes in physical activity and function with transition to retirement living: A pilot study. Can J Aging. 2016;35(4):526–32. 10.1017/S0714980816000593 2016-59260-011. 27917755

[147] Thiel A, Seiberth K, Mayer J. Why does theory matter? Reflections on an apparently self-evident question in sport sociology. European Journal for Sport and Society. 2018;15(1):1–4.

[148] Flake JK, Fried EI. Measurement schmeasurement: Questionable measurement practices and how to avoid them [Preprint]. 2019 10.31234/osf.io/hs7wm.

[149] Sallis JF. Age-related decline in physical activity: A synthesis of human and animal studies. Med Sci Sport Exer. 2000;32(9):1598–600. 10.1097/00005768-200009000-00012 10994911

[150] Bauman AE, Reis RS, Fallis JF, Wells JC, Loos RJF, Martin BW. Correlates of physical activity: Why are some people physically active and others not? Lancet. 2012;380:258–71. 10.1016/S0140-6736(12)60735-1 22818938

[151] Coughlin SS. Recall bias in epidemiologic studies. J Clin Epidemiol. 1990;43(1):87–91. 10.1016/0895-4356(90)90060-3 2319285

[152] Adams SA, Matthews CE, Ebbeling CB, Moore CG, Cunningham JE, Fulton J, et al The effect of social desirability and social approval on self-reports of physical activity. Am J Epidemiol. 2005;161(4):389–98. 10.1093/aje/kwi054 15692083 PMC2958515

