# Supplementary material for: The impact of life events and transitions on physical activity: A scoping review
Source: PLoS One. 2020 Jun 22;15(6):e0234794. doi: 10.1371/journal.pone.0234794 (PMC7307727; doi:10.1371/journal.pone.0234794)
Supplement: S1 Table — (DOCX) [file pone.0234794.s001.docx]

**S1 Table. Study characteristics of the 107 included studies arranged by the assessed life event and transition categories**

| **Author(s)/Year/ Country [Citation No.]** | **Life Events/Transitions Covered** | **Study Design** | | **Sample^[[1]](#footnote-1)^** | | **Age (yrs.)^[[2]](#footnote-2)^** | **PA Assessment Tools** | **PA Domain** | | | | | | | | **PA Intensity** | | | | |
| --- | --- | --- | --- | --- | --- | --- | --- | --- | --- | --- | --- | --- | --- | --- | --- | --- | --- | --- | --- | --- |
|  |  |  |  |  |  |  |  | LTPA | OPA | Total PA | Walking/daily PA | Commuting/transport | Domestic activity | School activity | Not specified | LPA | MPA | VPA | MVPA | Not specified |
| ***Multiple events/transitions*** |  |  | |  | |  |  |  |  |  |  |  |  |  |  |  |  |  |  |  |
| Bell & Lee (2005) Australia [70] | Residential independence, employment status, relationship status (getting married, cohabitation), motherhood | Prospective longitudinal 4 yrs. follow-up | | n = 8,545 [F: 100%] | | 18-23 [20.7 ± 1,48] | Q: Australian National Heart Foundation Risk Factor Prevalence Study & The Active Australia Survey | ● |  |  | ● |  |  |  |  |  | ● | ● | ● |  |
| Brown & Trost (2003) Australia [71] | Getting married, having the first or another baby, divorce, becoming a single parent, return to study, beginning paid work, changing work | Prospective longitudinal 4 yrs. follow-up | | n = 7,281 (F: 100%) | | 18-23 | Q: Australian National Heart Foundation Risk Factor Prevalence Study & The Active Australia Survey | ● |  |  | ● |  |  |  |  |  | ● | ● | ● |  |
| Brown et al. (2009) Australia [40] | Multitude of cohort-specific life events for young, mid-age, and old women | Prospective longitudinal 3 yrs. follow-up | | Total sample: n = 22,535 [F: 100%]  Young cohort: n = 7,173 [F: 100%]  Mid-age cohort: n = 8,762 [F: 100%]  Older cohort: n = 6,600 [F: 100%] | | n.r. 22-27 51-56 73-78 | Q: n.r. | ● |  |  | ● | ● |  |  |  |  | ● | ● | ● |  |
| Colley et al. (2019) United Kingdom [109] | Getting married, becoming a parent, retirement, onset of disabilities or ill health, death of others | Retrospective | | n = 27 [F: 70.4%] | | 66-91 | Interview  (individual and group) | ● |  |  |  |  |  |  |  |  |  |  |  | ● |
| Dai et al. (2014) Canada [77] | Changes in sociodemographic factors (income, education, employment status, martital status), changes in lifestyle and psychological factors (smoking, alcohol consumption, happiness, social support), changes in physical status (BMI, health status, body pain, long-term disability, chronic diseases) | Prospective longitudinal biannual follow-ups (over a maximum of 8 cycles) | | n = 12,901 [F: n.r.] | | n.r. | Q: n.r. | ● |  |  |  |  |  |  |  |  |  |  |  | ● |
| **Author(s)/Year/ Country [Citation No.]** | **Life Events/Transitions Covered** | | **Study Design** | | **Sample** | **Age (yrs.)** | **PA Assessment Tools** | **PA Domain** | | | | | | | | **PA Intensity** | | | | |
|  |  |  |  |  |  |  |  | LTPA | OPA | Total PA | Walking/daily PA | Commuting/transport | Domestic activity | School activity | Not specified | LPA | MPA | VPA | MVPA | Not specified |
| Gao et al. (2019) Netherlands [79] | Childbirth, getting a job, starting to work, stopping to work | Prospective longitudinal  2 yrs. follow-up | | n = 1,185 [F: 53.9%] | | n.r. | Travel diary  (3 days) |  |  |  | ● | ● |  |  |  |  |  |  |  | ● |
| Hull et al. (2010) USA [124] | Marriage, cohabitation, and parenthood | Prospective longitudinal 2 yrs. Follow-up | | Eligible: n = 646 [F: 52%]  Final analyses: n = 638 [F: 52%] | | 24.3 ± 1.1 24.0 ± 1.1 | Q: Past Year LTPAQ | ● |  |  |  |  |  |  |  |  |  |  |  | ● |
| Kenter et al. (2014) Netherlands [80] | Changes in physical mental health of oneself or the partner, changes in the social network, changes in employment status or in other self-defining activities, changes in sense of security, changes in living surroundings | | Retrospective | | n = 17 [F: 58.8%] | 60-82 | Interview (individual) | ● |  |  | ● | ● | ● |  |  |  |  |  |  | ● |
| Koeneman et al. (2012) Netherlands [95] | Retirement, widowhood | | Prospective longitudinal 3 yrs. Follow-up | | Total sample widowhood: n = 1,460 [F: 43%]  Still married at follow-up: n = 1,324 [F: 41%]  Widowed at follow-up: n = 136 [F: 68%]  Total sample retirement: n = 186 [F: 33%]  Retired at follow-up: n = 65 [F: 23%]  Employed at follow-up: n = 121 [F: 39%] | 67.4 ± 7.9 66.9 ± 7.8 71.7 ± 7.8 58.7 ± 2.6 58.1 ± 1.7 59.0 ± 2.9 | Q: LAPAQ | ● |  | ● | ● |  | ● |  |  |  |  |  | ● |  |
| Kostamo et al. (2019) Finland [115] | Health problems, educational transitions, changes in residence, (not) being supported | | Retrospective | | n = 115 | 15-24 | Critical Incident Technique | ● |  |  | ● | ● | ● | ● |  |  |  |  |  | ● |
| Larouche et al. (2012) Canada [58] | Puberty and associated entry into secondary school, beginning of postsecondary education, entry into the labor market, parenthood | | Retrospective | | n = 86 [F: 51.2%] | 44 ± 1.2 | Interview (individual) | ● | ● |  |  | ● | ● |  |  |  |  |  |  | ● |
| Miller et al. (2018) USA [59] | Having a first child, getting married, beginning or leaving post-secondary school, leaving parental house, beginning to work more than 20 hours/week | | Prospective longitudinal 15 yrs. Follow-up (4 assessments) | | Baseline: n = 4,746 [F: 49.8%]  Follow-up 1: n = 2,516 [F: 55.1%]  Follow-up 2: n = 2,287 [F: 55.0%]  Follow-up 3: n = 1,830 [F: 56.9%] | 11-18 [14.9 ± 1.7] 15-23 [19.4 ± 1.7] 19-31 [25.3 ± 1.6] 25-36 [31.1 ± 1.6] | Q: mod. GLTEQ |  |  |  |  |  |  |  | ● | ● | ● | ● | ● |  |

| **Author(s)/Year/ Country [Citation No.]** | **Life Events/Transitions Covered** | | **Study Design** | **Sample** | **Age (yrs.)** | **PA Assessment Tools** | **PA Domain** | | | | | | | | **PA Intensity** | | | | |
| --- | --- | --- | --- | --- | --- | --- | --- | --- | --- | --- | --- | --- | --- | --- | --- | --- | --- | --- | --- |
|  |  |  |  |  |  |  | LTPA | OPA | Total PA | Walking/daily PA | Commuting/transport | Domestic activity | School activity | Not specified | LPA | MPA | VPA | MVPA | Not specified |
| Paluch et al. (2017) USA [72] | Changing job, quitting/being fired from job, starting first job, graduation, leaving parent’s home, moving, going on welfare, starting a motgage, being arrested/going to jail, death of friend/family, serious injury or illness, starting/ending a relationship, engagement, marriage, pregnancy, divorce | | Prospective longitudinal 1 yr. follow-up (4 assessments) | n = 404 [F: 51.7%] | 21-35 [27.8 ± 3.7] | Accelerometry (10 days) |  |  | ● |  |  |  |  |  | ● | ● | ● | ● |  |
| Richards et al. (2019) USA [83] | Becoming married, becoming divorced, retirement, losing job, death of a child, death of a parent, death of spouse, death of a close friend, moving, becoming a caregiver | | Prospective longitudinal  3-25 yrs. Follow-up (1-4 assessments) | n = 3,617 [F: 62%] | 54 ± 17.62 | Q: n.r. | ● |  |  | ● |  | ● |  |  |  |  |  |  | ● |
| Simons et al. (2015) Australia [67] | Postgraduation (high school), moving out of home, employment status, education status | | Prospective longitudinal 2 yrs. Follow-up (3 assessements) | Follow-Up 1: n = 440 [F: 49.1%] Follow-Up 2: n = 374 [F: 49.5%] | 17.6 ± 0.6 17.5 ± 0.3 | Q: MLTPAQ | ● |  |  |  |  |  |  |  |  |  |  |  |  |
| Van Houten et al. (2017) Netherlands [78] | Beginning to work, starting to live on one’s own, starting cohabitation or getting married, birth of first child | | Retrospective | n = 3,540 [F: n.r.] | 18-35 | Q: n.r. | ● |  |  |  |  |  |  |  |  |  |  |  | ● |
| Van Houten et al. (2019) Netherlands [76] | Leaving full time education, beginning to work, entering an intimate relationship, starting cohabitation, getting married, birth of first child | | Prospective longitudinal  4 yrs. Follow-up | n = 2,317 [F: 54.3%] | 14-49 [31.7 ± 9.3] | Q: n.r. | ● |  |  |  |  |  |  |  |  |  |  |  | ● |
| ***Education-related events/transitions*** |  |  | |  |  |  |  |  |  |  |  |  |  |  |  |  |  |  |  |
| Barr-Anderson et al. (2017) USA [55] | Transition from elementary to middle school | Prospective longitudinal  2 yrs. follow-up (3 assessments) | | n = 643 [F: 54.1%] | n.r. | Accelerometry  (7 days) |  |  | ● |  |  |  |  |  | ● | ● | ● |  |  |

| **Author(s)/Year/ Country [Citation No.]** | **Life Events/Transitions Covered** | | **Study Design** | **Sample** | **Age (yrs.)** | **PA Assessment Tools** | **PA Domain** | | | | | | | | **PA Intensity** | | | | |
| --- | --- | --- | --- | --- | --- | --- | --- | --- | --- | --- | --- | --- | --- | --- | --- | --- | --- | --- | --- |
|  |  |  |  |  |  |  | LTPA | OPA | Total PA | Walking/daily PA | Commuting/transport | Domestic activity | School activity | Not specified | LPA | MPA | VPA | MVPA | Not specified |
| Bray & Born (2004) Canada [61] | Transition from high school to university | Retrospective | | n = 145 [F: 73.1%] | 18-19 [18.14 ± 0.42] | Q: Youth Risk Behavior Survey (1995) & National College Health Risk Behavior Survey (1995) | ● |  |  |  |  |  |  |  |  |  | ● |  |  |
| Bray (2007) Canada [60] | Transition from high school to university | Retrospective | | n = 127 [F: 68.5%] | 17-21 [17.76 ± 0.54] | Q: MAQ-A | ● |  |  | ● |  |  |  |  |  | ● | ● |  |  |
| Cooper et al. (2012) United Kingdom [46] | Transition from primary to secondary school | Prospective longitudinal 1 yr. follow-up | | Baseline: n = 1,307 [F: n.r.]  Final analyses: n = 500 [F: n.r.] | 11.0 ± 0.4 n.r. | Q: n.r. Accelerometry  (7 days) |  |  | ● |  |  |  |  |  |  |  |  | ● |  |
| Crane et al. (2018) Canada [45] | Transition from kindergarten to elementary school | Prospective longitudinal and cross-sectional  follow-up time n.r. | | Longitudinal sample: n = 21 [F: 50.4%] | n.r. | Accelerometry  (4 days) |  |  | ● |  |  |  |  |  |  |  |  | ● |  |
| Deforche et al. (2015) Belgium [69] | Transition from high school to university | Prospective longitudinal  1.5 yrs. follow-up | | n = 291 [F: 66.7%] | 17.2 ± 0.5 | Q: FPAQ | ● |  |  | ● | ● |  |  |  |  |  |  |  | ● |
| De Meester et al. (2014) Belgium [47] | Transition from primary to secondary school | Prospective longitudinal 2 yrs. Follow-up | | Baseline: n = 736 [F: n.r.]  Final analyses: n = 420 [F: 49.5%] | 10-13 [11.1 ± 0.5] 13.4 ± 0.6 | Q: FPAQ Accelerometry  (7 days) Pedometry  (7 days) |  |  | ● |  | ● |  | ● |  |  |  |  | ● |  |
| D’Haese et al. (2015) Belgium [48] | Transition from primary to secondary school | Prospective longitudinal 2 yrs. Follow-up | | Baseline: n = 736 [F: n.r.]  Final analyses: n = 321 [F: 48.9%] | 10-13 [11.1 ± 0.5] 13.4 ± 0.6 | Q: FPAQ Accelerometry  (7 days) Pedometry  (7 days) | ● |  | ● |  | ● |  | ● |  |  |  |  | ● |  |
| Diehl & Hilger (2016) Germany [62] | Transition from high school to university | Retrospective | | n = 689 [F: 69.5%] | 22.7 ± n.r | Q: n.r. |  |  |  |  |  |  |  | ● |  |  |  |  | ● |
| Garcia et al. (1998) USA [49] | Transition from elementary to junior high school | Prospective longitudinal 1 yr. follow-up | | n = 132 [F: 57.6%] | n.r. | Q: CAAL | ● |  |  |  |  |  |  |  |  |  |  |  | ● |
| Han et al. (2008) USA [64] | Transition from high school to university | Prospective longitudinal 1 yr. follow-up | | n = 69 [F: 100%] | 18-21 [18.2 ± 0.4] | Q: MAQ-A | ● |  |  |  |  |  |  |  |  | ● | ● | ● |  |
| Jago et al. (2012) United Kingdom [50] | Transition from primary to secondary school | Prospective longitudinal 1 yr. follow-up | | Baseline: n = 1,300 [F: n.r.]  Final analyses: n = dependent on analysis | 10-11 [10.95 ± 0.4]^[[3]](#footnote-3)^ | Accelerometry  (7 days) | ● |  |  |  |  |  |  |  |  |  |  | ● |  |

| **Author(s)/Year/ Country [Citation No.]** | **Life Events/Transitions Covered** | | **Study Design** | **Sample** | **Age (yrs.)** | **PA Assessment Tools** | **PA Domain** | | | | | | | | **PA Intensity** | | | | |
| --- | --- | --- | --- | --- | --- | --- | --- | --- | --- | --- | --- | --- | --- | --- | --- | --- | --- | --- | --- |
|  |  |  |  |  |  |  | LTPA | OPA | Total PA | Walking/daily PA | Commuting/transport | Domestic activity | School activity | Not specified | LPA | MPA | VPA | MVPA | Not specified |
| Jáuregui et al. (2011) Mexico [41] | Transition from kindergarten to elementary school | Prospective longitudinal 3 yrs. Follow-up (3 assessments) | | Baseline: n = 320 [F: n.r.] Final analyses: n = 217 [F: 56.7%] | 5-6 n.r. | Accelerometry  (5 days) | ● |  |  |  | ● |  | ● |  |  |  |  | ● |  |
| Li et al. (2016) USA [73] | Postgraduation (high school) | Prospective longitudinal 4 yrs. Follow-up (annual assessments) | | n = 2,659 [F: 55%] | 16.2 ± 0.02 | Q: Youth Risk Behavior Surveillance & Health Behavior in School-Aged Children Survey | ● |  |  |  | ● |  | ● |  |  |  | ● | ● |  |
| Marks et al. (2015) Australia [54] | Transition from primary school to secondary school | Prospective longitudinal  5-8 mo. follow-up | | n = 243 [F: 59.7%] | 11-13 [12.2 ± n.r.] | Q: PAQ-C & ABAKQ | ● |  |  |  | ● |  |  |  | ● |  |  | ● |  |
| Molina-García et al. (2015) Spain [74] | Postgraduation (high school) | Prospective longitudinal 1 yr. follow-up | | Baseline: n = 244 [F: 58.6%]  Final analyses: n = 111 [F: 49.5%] | 17.6 ± 0.7 18.6 ± 0.7 | Q: GPAQ (Spanish) | ● | ● | ● |  | ● |  |  |  |  |  |  |  |  |
| Oja & Jürimäe (2001) Estonia [42] | Transition from preschool to school | Prospective longitudinal 2 yrs. Follow-up (4 assessments) | | n = 294 [F: 45.2%] | 6 | Q: Harro | ● |  |  |  |  |  | ● |  |  |  |  |  | ● |
| Owens et al. (2013) United Kingdom [75] | Postgraduation (out of compulsory education) | Prospective longitudinal 1 yr. follow-up | | Baseline: n = 2,204 [F: 45.8%]  Sample 1: n = 663 [F: 45.4%]  Sample 2: n = 834 [F: 46.4%] | 14-17  n.r.  n.r. | Q: MAQ-A | ● |  |  |  |  |  |  |  |  |  |  | ● |  |
| Parra-Salídas et al. (2019) Chile [68] | Transition from high school to university | Retrospective | | n = 1,288 [F: 52.3%] | 22.7 ± 5.8 | Q: n.r. |  |  |  |  | ● |  |  |  |  |  |  |  | ● |
| Pate et al. (2019) USA [52] | Transition from elementary to middle school | Prospective longitudinal  3 yrs. follow-up (3 assessments) | | n = 828 [F: 53.9%] | 10.6 ± 0.5 | Accelerometry  (7 days) |  |  |  |  |  |  |  | ● | ● | ● | ● |  |  |
| Pullman et al. (2009) Canada [65] | Transition from high school to university | Prospective longitudinal 9 mo. follow-up (3 assessments) | | n = 108 [F: 0%] | 17-20 [18.5 ± 0.1] | Q: National Longitudinal Survey of Children and Youth (2006) & National Youth Risk Behavior Survey (1990) | ● |  |  |  |  |  |  |  |  | ● | ● |  |  |
| Ridley & Dollman (2019) Australia [53] | Transition from primary school to secondary school | Prospective longitudinal  1 yr. follow-up | | Transition cohort: n = 61 [F: 100%] Secondary cohort: n = 57 [F: 100%] | n.r. | Q: PAQ-C | ● |  |  |  |  |  | ● |  |  |  |  |  | ● |
| Rutten et al. (2014) Belgium [51] | Transition from elementary to secondary school | Prospective longitudinal 2 yrs. Follow-up | | n = 472 [F: 55%] | 10.97 ± 0.41 | Q: PAQ-C Pedometry  (7 days) | ● |  |  |  |  |  | ● |  |  |  |  | ● |  |
| Shull et al. (2019) USA [57] | Transition from middle to high school | Prospective longitudinal  2 yrs. Follow-up | | n = 306 [F: 60%] | 12-17 [12.5 ± 0.5] | Q: n.r. Accelerometry  (7 days) | ● |  | ● |  |  |  |  |  |  |  |  | ● |  |
| **Author(s)/Year/ Country [Citation No.]** | **Life Events/Transitions Covered** | | **Study Design** | **Sample** | **Age (yrs.)** | **PA Assessment Tools** | **PA Domain** | | | | | | | | **PA Intensity** | | | | |
|  |  |  |  |  |  |  | LTPA | OPA | Total PA | Walking/daily PA | Commuting/transport | Domestic activity | School activity | Not specified | LPA | MPA | VPA | MVPA | Not specified |
| Sigmund et al. (2009) Czech Republic [43] | Transition from preschool to school | Prospective longitudinal 1 yr. follow-up | | Baseline: n = 208 [F: 51%]  Final analyses: n = 176 [F: 47.7%] | 5-7 n.r. | Accelerometry  (7 days) Pedometry  (7 days) | ● |  | ● |  |  |  | ● |  |  |  |  |  | ● |
| Taylor et al. (2013) New Zealand [44] | Transition from preschool to school | Prospective longitudinal  4 yrs. Follow-up (6 assessments) | | n = 242 [F: 43.4%] | 3 | Accelerometry (at least 5 days) |  |  | ● |  |  |  |  |  | ● | ● | ● |  |  |
| Taymoori et al. (2011) Iran [56] | Transition from middle to high school | Prospective longitudinal 3 yrs. Follow-up | | Baseline: n = 1,073 [F: 52%]  Final analyses: n = 883 [F: 49%] | 12-15 [14.37 ± 1.6] 15-18 [16.42 ± 1.73] | Q: mod. CAAL |  |  | ● |  |  |  |  |  |  |  |  |  | ● |
| Ullrich-French et al. (2013) USA [66] | Transition from high school to university | Prospective longitudinal (Baseline during late spring/summer berfore university; follow-up in early November of the fall semester) | | n = 238 [F: 70%] | 18-20 [18.65 ± 0.32] | Q: one item from GLTEQ | ● |  |  |  |  |  |  |  |  |  | ● |  |  |
| Van Dyck et al. (2015) Belgium [63] | Transition from high school to university | Prospective longitudinal 2 yrs. Follow-up | | n = 291 [F: 66.7%] | 17.2 ± 0.5 | Q: FPAQ | ● |  |  |  | ● |  |  |  |  |  |  |  | ● |
| ***Employment-related events/transitions*** |  |  | |  |  |  |  |  |  |  |  |  |  |  |  |  |  |  |  |
| Barnett et al. (2014) United Kingdom [84] | Retirement | Prospective longitudinal ~ 7.5 yrs. Follow-up | | Retired at follow-up: n = 785 [F: 52.9%]  Employed at follow-up: n = 2,549 [F: 51.8%] | 59.7 ± 4.7 53.0 ± 5.1 | Q: EPAQ2 | ● | ● | ● | ● |  | ● |  |  |  |  |  |  | ● |
| Berger et al. (2005) United Kingdom [85] | Retirement | Prospective longitudinal 4-5 years follow-up | | n = 699 [F: n.r.] | 60 ± n.r. | Interview (individual) | ● | ● |  | ● |  | ● |  |  |  | ● | ● |  |  |
| Chung et al. (2009) USA [86] | Retirement | Prospective longitudinal 6 yrs. Follow-up (4 assessments) | | n = 11,469 [F: 52.9%]  n = 34,019 observations  (retired: n = 11,542; employed: n = 22,477) | 60.3 ± 4.78 | Q: n.r. | ● | ● |  |  |  | ● |  |  |  |  | ● |  |  |
| Ding et al. (2016) Australia [87] | Retirement | Prospective longitudinal 3.3 ± 0.9 yrs. Follow-up | | Retired at follow-up: n = 3,106 [F: 49.3%]  Employed at follow-up: n = 24,151 [F: 50.7%] | 62.4 ± 6.7 54.3 ± 6.2 | Q: The Active Australia Survey |  |  | ● | ● |  |  |  |  |  | ● | ● | ● |  |
| Evenson et al. (2002) USA [88] | Retirement | Prospective longitudinal 6 yrs. Follow-up | | Total sample: n = 7,782 [F: 49.8%]  Retired at follow-up: n = 2,293 [F: 44%]  Employed at follow-up: n = 5,489 [F: 52.2%] | 45-64 n.r. n.r. | Q: Baecke | ● | ● |  | ● | ● |  |  |  |  |  |  |  | ● |
| Feng et al. (2016) USA [89] | Retirement | Prospective longitudinal 2 yrs. Follow-up | | n = 5,754 [F: n.r.] n = 24,224 person-years | 50-75 | Q: n.r. | ● | ● |  | ● |  | ● |  |  | ● | ● | ● |  |  |
| Henkens et al. (2008) Netherlands [90] | Retirement | Prospective longitudinal 6 yrs. Follow-up | | n = 1,604 [F: n.r.] | 50-64 [54.8 ± 2.8] | Q: n.r. |  |  |  |  |  |  |  | ● |  |  |  |  | ● |
| Henning et al. (2020) Sweden [108] | Retirement | Prospective longitudinal  4 yrs. Follow-up (2-4 assessments) | | n = 1,033 [F: 56.8%] | 60-66 [63.34 ± 1.64] | Q: n.r. | ● |  |  | ● |  | ● |  |  |  |  |  |  | ● |

| **Author(s)/Year/ Country [Citation No.]** | **Life Events/Transitions Covered** | | **Study Design** | **Sample** | **Age (yrs.)** | **PA Assessment Tools** | | **PA Domain** | | | | | | | | | | | | | | | | **PA Intensity** | | | | | | | | | |
| --- | --- | --- | --- | --- | --- | --- | --- | --- | --- | --- | --- | --- | --- | --- | --- | --- | --- | --- | --- | --- | --- | --- | --- | --- | --- | --- | --- | --- | --- | --- | --- | --- | --- |
|  |  |  |  |  |  |  |  | LTPA | | OPA | | Total PA | | Walking/daily PA | | Commuting/transport | | Domestic activity | | School activity | | Not specified | | LPA | | MPA | | VPA | | MVPA | | Not specified | |
| Holstila et al. (2017) Finland [91] | Retirement | Prospective longitudinal 10-12 yrs. Follow-up (3 assessments) | | Total sample: n = 2,902 [F: 79.6%]  Retired in first phase: n = 851 [F: 75.7%]  Retired in second phase: n = 948 [F: 81.3%]  Employed at follow-up: n = 1,103 [F: 81.1%] | 50-60 [54.4 ± 3.72] 58.5 ± 2.35 54.9 ± 1.8 51 ± 2.14 | Q: n.r. | | ● | |  | |  | | ● | |  | |  | |  | |  | | ● | | ● | | ● | | ● | |  | |
| Jones et al. (2018a) USA [92] | Retirement | Prospective longitudinal ~ 10 yrs. Follow up (4 assessments) | | Total sample: n = 928 [F: 54%] No recreational walking: n = 136 [F: 60%] No transport walking: n = 41 [F: 41%] | 56-64  55-65 56-64 | Q: n.r. | |  | |  | |  | | ● | | ● | |  | |  | |  | |  | |  | |  | |  | | ● | |
| Jones et al. (2018b) USA [93] | Retirement | Prospective longitudinal 9 years (median) follow-up (5 assessments) | | Total sample: n = 4,091 [F: 56%]  Retired at follow-up: n = 1,012 [F: 53%]  Employed at follow-up: n = 3,079 [F: 57%] | 45-84 [57.3 ± 8.9] 58.4 ± 6.9 56.9 ± 9.4 | Q: MESA PAQ | | ● | | ● | |  | | ● | |  | | ● | |  | |  | |  | | ● | | ● | | ● | |  | |
| Kämpfen & Maurer (2016) USA [94] | Retirement | Prospective longitudinal 6 yrs. Follow-up (4 assessments) | | n = 13,491 [F: n.r.]  n = 47,336 observations (on average 3.5 observations per individual) | 50-80 [65.3 ± n.r.] | Q: n.r. | ● | |  | |  | | ● | |  | | ● | |  | |  | | ● | | ● | | ● | |  | |  | |  |
| Kirk & Rhodes (2012) Canada [81] | Career transitions of academic professors | Retrospective | | n = 267 [F: 57.3%] | 25-44 [38.3 ± 6.48] | Q: mod. GLTEQ | ● | |  | |  | |  | |  | |  | |  | |  | |  | |  | |  | | ● | |  | |  |
| Lahti et al. (2011) Finland [96] | Retirement | Prospective longitudinal 5-7 yrs. Follow-up | | n = 6,706 [F: 81.3%] | 40-60 | Q: n.r. | ● | |  | |  | | ● | | ● | |  | |  | |  | |  | | ● | | ● | |  | |  | |  |
| Littman et al. (2015) USA [82] | Discharge from military | Prospective longitudinal ~ 6 years follow-up (3 assessments) | | Total sample: n = 28,866 [F: n.r.]  Discharge group: n = 3,782 [F: 25.6%] | n.r. n.r. | Q: n.r. | ● | |  | |  | | ● | |  | | ● | |  | |  | |  | | ● | | ● | | ● | |  | |  |
| McDonald et al. (2015) United Kingdom [97] | (Anticipated) retirement | Retrospective | | n = 28 [F: 53.6%] | 55-67 [61 ± 2.79] | Interview (individual) | ● | | ● | |  | |  | | ● | | ● | |  | |  | |  | |  | |  | |  | | ● | |  |
| McDonald et al. (2017) United Kingdom [98] | Retirement | Prospective longitudinal  2-7 mo. (daily assessments/n-of-1 design) | | n = 7 [F: 71.4%] | 55-76 [62.7 ± 6.5] | Q: EPIC PAQ Accelerometry |  | | ● | | ● | |  | |  | |  | |  | |  | |  | |  | |  | |  | | ● | |  |
| Menai et al. (2014) France [99] | Retirement | Prospecive longitudinal 6 yrs. Follow-up | | Total sample: n = 2,841 [F: 48.9%] Retired at baseline: n = 824 [F: 36%]  Retired at follow-up: n = 891 [F: 44.4%]  Employed at follow-up: n = 1,126 [F: 61.7%] | n.r. 62.3.1 ± 3.3 57.1 ± 3.5 53.1 ± 3.2 | Q: MAQ (French) | ● | | ● | |  | | ● | |  | | ● | |  | |  | |  | | ● | | ● | |  | |  | |  |
| Oshio & Kan (2017) Japan [100] | Retirement | Prospective longitudinal 10 yrs. Follow-up (annual assessments) | | n = 9,283 [F: 52.2%]  n = 54,113 observations | 50-59 | Q: n.r. | ● | |  | |  | |  | |  | |  | |  | |  | |  | |  | |  | | ● | |  | |  |
| Schönbach et al. (2017) Germany [101] | Retirement | Prospective longitudinal 20 yrs. Follow-up (annual assessments) | | n = 3,233 [F: 44.4%]  n = 20,597 observations Non-migrant background: n = 2,664 [F: 44.4%]  Migrant background: n = 569 [F: 44.7%] | 55-75  61.63 ± 2.85^[[4]](#footnote-4)^ 61.51 ± 2.95 | Q: n.r. | ● | |  | |  | |  | |  | |  | |  | |  | |  | |  | |  | |  | | ● | |  |
| Sjösten et al. (2012) France [102] | Retirement | Prospective longitudinal 9 yrs. Follow-up (annual assessments) | | Sample 1: n = 2,711 [F: 37.2%];  n = 19,673 observations-years Sample 2: n = 3,812 [F: 24.7%] | 50-66 [58 ± 2.4]^5^ 48-63 [56 ± 2.4] | Q: n.r. | ● | |  | |  | | ● | |  | |  | |  | |  | |  | |  | |  | |  | | ● | |  |
| **Author(s)/Year/ Country [Citation No.]** | **Life Events/Transitions Covered** | **Study Design** | | **Sample** | **Age (yrs.)** | **PA Assessment Tools** | | **PA Domain** | | | | | | | | | | | | | | | | **PA Intensity** | | | | | | | | | |
|  |  |  |  |  |  |  |  | LTPA | | OPA | | Total PA | | Walking/daily PA | | Commuting/transport | | Domestic activity | | School activity | | Not specified | | LPA | | MPA | | VPA | | MVPA | | Not specified | |
| Slingerland et al. (2007) Netherlands [103] | Retirement | Prospective longitudinal 13 yrs. Follow-up | | Total sample: n = 971 [F: 25%]  Retired at follow-up: n = 684 [F: 23%]  Employed at follow-up: n = 287 [F: 29%] | 40-65 [50.0 ± 5.3] 40-65 [52.4 ± 4.3] 40-65 [44.4 ± 3.0] | Q: SQUASH (only at follow-up) | ● | |  | |  | |  | | ● | |  | |  | |  | |  | |  | |  | |  | | ● | |  |
| Sprod et al. (2017) Australia [104] | Retirement | Prospective longitudinal (assessments at preretirement and 3, 6 and 12 mo. After retirement) | | n = 124 [F: 51%] | 50.1-78.6 [62.3 ± 4.3] | Q: MARCA |  | |  | |  | |  | |  | |  | |  | | ● | | ● | | ● | | ● | |  | |  | |  |
| Stenholm et al. (2016) Finland [105] | Retirement | Prospective longitudinal 12 yrs. Follow-up (4 assessments) | | Total sample: n = 9,488 [F: 80%]  Statutory retirement: n = 5,770 [F: 79.9%]  Part-time retirement: n = 1,587 [F: 78.4%]  Disability retirement: n = 2,131 [F: 83.6%] | n.r. 61.92 ± 2.00^5^ 58.93 ± 2.02 55.38 ± 5.80 | Q: n.r. | | ● | |  | | ● | | ● | | ● | |  | |  | |  | |  | | ● | | ● | |  | |  | |
| Touvier et al. (2010) France [106] | Retirement | Prospective longitudinal 3 yrs. Follow-up | | Total sample: n = 1,389 [F: 49.8%]  Retired at follow-up: n = 248 [F: 43.6%]  Employed at follow-up: n = 1,141 [F: 51.1%] | 45-64 n.r. n.r. | Q: MAQ (French) | | ● | | ● | |  | | ● | |  | |  | |  | |  | | ● | | ● | | ● | |  | |  | |
| Van Dyck et al. (2016) Belgium [107] | Retirement | Prospective longitudinal 2 yrs. Follow-up | | n = 446 [F: 47.4%] Already retired at baseline: n = 340 [F: 49.1%]  Retired at follow-up: n = 105 [F: 41.9%] | 62.4 ± 2.2 62.8 ± 2.0 61.3 ± 2.6 | Q: IPAQ | | ● | | ● | |  | | ● | | ● | | ● | |  | |  | |  | | ● | |  | | ● | |  | |
| ***Health-related events/transitions*** |  |  | |  |  |  | |  | |  | |  | |  | |  | |  | |  | |  | |  | |  | |  | |  | |  | |
| Beal et al. (2016) USA [121] | Onset of menarche | Prospective longitudinal 3 yrs. Follow-up (3 assessments & additional phone interviews) | | n = 262 [F: 100%] | 11-19 [12.41 ± 1.25] | Q: PAQ-C | | ● | |  | |  | |  | |  | |  | |  | |  | |  | |  | |  | |  | | ● | |
| Blanchard et al. (2003) USA [110] | Cancer diagnosis | Retrospective | | n = 352 [F: 71%] | 59.6 ± 12.7 | Q: n.r. | | ● | |  | |  | |  | |  | |  | |  | |  | |  | |  | |  | |  | | ● | |
| Cerimagic et al. (2015) Australia [111] | Urological cancer diagnosis | Retrospective | | n = 50 [F: 10%] | n.r. | Interview (individual) | |  | |  | |  | |  | |  | |  | |  | | ● | |  | |  | |  | |  | | ● | |
| Clark et al. (2015) Finland [116] | Onset of impaired sleep | Prospective longitudinal ~ 8 yrs. Follow-up (3 assessments) | | n = 37,508 [F: 83%] n = 59,152 person observations | 18-69 [46 ± 9] | Q: n.r. | | ● | |  | |  | |  | |  | |  | |  | |  | |  | |  | |  | |  | | ● | |
| Duval et al. (2016) Canada [118] | Menopausal transition | Prospective longitudinal 5 yrs. Follow-up (annual assessments) | | n = 102 [F: 100%;] | 47-55 [49.9 ± 1.9] | Accelerometry  (7 days) | |  | |  | | ● | |  | |  | |  | |  | |  | |  | | ● | |  | |  | |  | |
| Humpel et al. (2007) Australia [112] | Cancer diagnosis | Retrospective | | Total sample: n = 657 [F: 81.4%] Cancer survivor group: n = 113 [F: 86.7%] Non-cancer group: n = 544 [F: 80.3%] | 46.0 ± 15.0 n.r. n.r. | Q: The Active Australia Survey | |  | |  | | ● | | ● | |  | |  | |  | |  | |  | | ● | | ● | |  | |  | |

| **Author(s)/Year/ Country [Citation No.]** | **Life Events/Transitions Covered** | **Study Design** | | **Sample** | **Age (yrs.)** | **PA Assessment Tools** | **PA Domain** | | | | | | | | **PA Intensity** | | | | |
| --- | --- | --- | --- | --- | --- | --- | --- | --- | --- | --- | --- | --- | --- | --- | --- | --- | --- | --- | --- |
|  |  |  |  |  |  |  | LTPA | OPA | Total PA | Walking/daily PA | Commuting/transport | Domestic activity | School activity | Not specified | LPA | MPA | VPA | MVPA | Not specified |
| Li et al. (2013) USA [114] | Disease and functional difficulties | Prospective longitudinal 6 yrs. Follow-up (4 assessments) | | n = 2,998-5,074 couples | F: 64.48 ± 8.89 M: 67.43 ± 9.20 | Q: mod. GLTEQ | ● |  |  | ● |  | ● |  |  | ● | ● | ● |  |  |
| Lovejoy et al. (2008) USA [119] | Menopausal transition | Prospective longitudinal 4 yrs. Follow-up (annual measurments) | | n = 129 [F: 100%] | n.r. | Accelerometry  (4 days) |  |  | ● |  |  |  |  |  |  |  |  |  | ● |
| Moilanen et al. (2012) Finland [120] | Menopausal transition | Prospective longitudinal 8 yrs. Follow-up | | n = 1,165 [F: 100%] | 37-56 [47.0 ± 5.2] | Q: n.r. | ● |  |  | ● |  | ● |  |  |  | ● | ● |  |  |
| Satia et al. (2004) USA [113] | Colon cancer diagnosis | Prospective longitudinal ~ 2 yrs. Follow-up | | Total sample: n = 737 [F: n.r.]  Cancer survivor group: n = 278 [F: 47.1%]  Control group: n = 459 [F: 47.1%] | 40-80 63.3 ± 10.2 64.9 ± 9.5 | Q: mod. PAR | ● |  |  |  |  |  |  |  |  |  |  |  | ● |
| Zhou et al. (2018) China [117] | Onset of various chronic diseases | Prospective longitudinal 6 yrs. Follow-up (6 assessments) | | n = 3,094 [F: n.r.]  n = 13,636 observations | 55-99 | Q: IPAQ | ● |  |  |  |  |  |  |  |  |  |  |  | ● |
| ***Relationship-related events/transitions*** |  |  | |  |  |  |  |  |  |  |  |  |  |  |  |  |  |  |  |
| Eng et al. (2005) USA [128] | Divorce/separation, widowhood, re-marriage | Prospective longitudinal 8 yrs. Follow-up (3 assessments) | | Total sample: n = 38,865 [F: 0%]  Married group: n = 36,285 [F: 0%]  Divorced group: n = 2,050 [F: 0%]  Widowed group: n = 530 [F: 0%] | 40-74 54.9 ± 9.8 51.4 ± 8.5 63.7 ± 8.2 | Q: n.r. | ● |  |  | ● |  |  |  |  |  |  |  |  | ● |
| Ezendam et al. (2019) Denmark [131] | Cancer diagnosis of partner | Prospective longitudinal  follow-up time n.r. | | Diagnosis of cancer: n = 672 [F: 55%] Cancer free: n = 5,534 [F: 55%] | 50-65 [57 ± 4.1] 50-66 [57 ± 4.1] | Q: n.r. | ● |  |  | ● |  |  |  |  |  |  |  |  | ● |
| Josefsson et al. (2018) Finland [123] | Marriage, cohabitation, divorce/separation, widowhood | Prospective longitudinal 12 yrs. Follow-up (4 assessments) | | n = 81,925 [F: 80.4%]  n = 327,700 person observations | 18-77 | Q: n.r. | ● |  |  | ● |  |  |  |  | ● | ● | ● |  |  |
| King et al. (1998) USA [125] | Marriage, divorce/separation | Prospective longitudinal 10 yrs. Follow-up (5 assessments) | | n = 558 [F: 54.1%] | 25-74 [44.0 ± 15.1] | Interview (individual) | ● | ● |  |  |  |  |  |  |  |  |  |  | ● |
| Kutob et al. (2017) USA [126] | Marriage (-like relationship), divorce/separation | Prospective longitudinal 3 yrs. Follow-up | | n = 79.094 [F: 100%] | 50-79 | Q: n.r. | ● |  |  | ● |  |  |  |  |  | ● | ● |  |  |
| Lee et al. (2005) USA [127] | Widowhood, divorce, marriage | Prospective longitudinal 4 yrs. Follow-up | | n = 80,264 [F: 100%] | 46-71 | Q: n.r. | ● |  |  |  |  |  |  |  |  |  |  |  | ● |
| Mueller & Shaikh (2018) 19 European countries [132] | Retirement of spouse | Prospective longitudinal  follow-up time n.r. | | n = 23,598 [F: n.r.] | 45-91 [63.35 ± 5.9] | Q: n.r. |  |  |  |  |  |  |  | ● |  | ● | ● |  |  |
| Salin et al. (2019) Finland [122] | Re-coupling, starting relationship, divorce | Prospective longitudinal  4 yrs. follow-up | | n = 1,994 [F: 57.4%] | 30-45 [37.8 ± 5] | Pedometry  (7 days) |  |  |  | ● |  |  |  |  |  |  |  |  | ● |
| Stahl & Schulz (2014) USA [129] | Widowhood | Prospective longitudinal 8 yrs. follow-up (3 assessments) | | Total sample: 792 [F: 77.5%] Widowed group: n = 396 [F: 77.5%]  Married group: n = 396 [F: 77.5%] | n.r. 72.81 ± 5.32 71.12 ± 3.96 | Q: MLTPAQ | ● |  |  | ● |  |  |  |  |  |  |  | ● |  |
| **Author(s)/Year/ Country [Citation No.]** | **Life Events/Transitions Covered** | | **Study Design** | **Sample** | **Age (yrs.)** | **PA Assessment Tools** | **PA Domain** | | | | | | | | **PA Intensity** | | | | |
|  |  |  |  |  |  |  | LTPA | OPA | Total PA | Walking/daily PA | Commuting/transport | Domestic activity | School activity | Not specified | LPA | MPA | VPA | MVPA | Not specified |
| Wilcox et al. (2003) USA [130] | Widowhood, re-marriage | Prospective longitudinal 3 yrs. follow-up | | Baseline: n = 72,247 [F: 100%]  Final analyses: n = 55,724 [F: 100%] | 50-79 [64.09 ± 7.32] 50-79 | Q: n.r. | ● |  |  | ● |  |  |  |  |  | ● | ● |  |  |
| ***Family-related events/transitions*** |  |  | |  |  |  |  |  |  |  |  |  |  |  |  |  |  |  |  |
| Albright et al. (2005) USA [133] | Pregnancy, parenthood | Retrospective | | n = 79 [F: 100%] | 18-45 [31.8 ± 5.5] | Interview (group) Q: n.r. | ● |  |  |  |  |  |  |  |  | ● |  |  |  |
| Cramp & Bray (2009) Canada [134] | Pregnancy, parenthood | Retrospective | | n= 309 [F: 100%] | 30.0 ± 4.0 | Q: MAQ | ● |  |  |  |  |  |  |  |  |  |  | ● |  |
| Grace et al. (2006) Canada [135] | Pregnancy, parenthood | Prospective longitudinal 17 mo. follow-up (2 assessments) | | Total sample: n = 243 [F: 100%]  Maternity group: n = 42 [F: 100%] | 39.5 ± 7.95 32.6 ± 4.2 | Q: HPLP II | ● |  |  |  |  |  |  |  |  | ● |  |  |  |
| Hamilton & White (2010) Australia [136] | Parenthood | Retrospective | | n = 40 [F: 52.5%] | 23-49 [35 ± n.r.] | Interview (group/ individual) | ● | ● |  | ● |  | ● |  |  |  | ● |  |  |  |
| Hinton & Olson (2001) USA [137] | Pregnancy | Prospective longitudinal ~ 2 yrs. follow-up | | n = 622 [F: 100%] | 28.8 ± 5.44 | Q: mod. GLTEQ | ● |  |  |  |  |  |  |  |  |  |  |  | ● |
| Hull et al. (2015) USA [138] | Parenthood | Prospective longitudinal 2- to 3 yrs. follow-up | | Baseline: n = 49 [F: 69%]  Final analyses: n = 49 [F: 69%] | 26.3 ± 1.1 28.9 ± 1.7 | Q: Past Year LTPAQ Interview | ● |  |  |  |  |  |  |  |  |  |  |  | ● |
| McIntyre & Rhodes (2009) Canada [139] | Parenthood | Retrospective | | n = 139 [F: 100%] | 25-34 [31.21 ± 2.75] | Q: GLTEQ | ● |  | ● |  |  |  |  |  | ● | ● | ● |  |  |
| Perales et al. (2015) Australia [140] | Parenthood | Prospective longitudinal 12 yrs. follow-up (annual assessments) | | n = 23,515 [F: 52.2%]  n = 132, 610 yearly observations [F: 53.3%] | n.r. | Q: HILDA Survey |  |  |  |  |  |  |  | ● |  |  |  | ● |  |
| Pereira et al. (2007) USA [141] | Pregnancy, parenthood | Prospective longitudinal ~ 1 yr. follow-up (3 assessments) | | Follow-up 1: n = 1,442 [F: 100%]  Follow-up 2: n = 1,242 [F: 100%] | 32.5 ± 4.5 | Q: mod. PASE | ● |  | ● | ● |  |  |  |  | ● | ● | ● |  |  |
| Rhodes et al. (2014) Canada [142] | Parenthood | Prospective longitudinal 1 yr. follow-up (3 assessments) | | Total sample: n = 314 [F: 50%]  No children group: n = 102 [F: 50%] Onset of first child group: n = 136 [F: 50 %]  Onset of second child group: n = 76 [F: 50%] | 25-40 28.46 ± 5.28 32.11 ± 4.83 33.32 ± 4.52 | Accelerometry  (7 days) |  |  | ● |  |  |  |  |  | ● |  |  | ● |  |
| Sjögren Forss & Stjernberg (2019) Sweden [145] | Pregnancy, parenthood | Prospective longitudinal  follow-up time n.r. (3 assessments) | | n = 235 [F: 52.3%] | Women: 31  Men: 33 | Q: n.r. | ● |  |  | ● |  | ● |  |  |  |  |  |  | ● |
| Symons Downs & Hausenblas (2004) USA [143] | Pregnancy, parenthood | Retrospective | | n = 74 [F: 100%] | 19-40 [31.3 ± 4.37] | Q: GLTEQ | ● |  |  |  |  |  |  |  | ● | ● | ● |  |  |

| **Author(s)/Year/ Country [Citation No.]** | **Life Events/Transitions Covered** | | **Study Design** | **Sample** | **Age (yrs.)** | **PA Assessment Tools** | **PA Domain** | | | | | | | | **PA Intensity** | | | | | |
| --- | --- | --- | --- | --- | --- | --- | --- | --- | --- | --- | --- | --- | --- | --- | --- | --- | --- | --- | --- | --- |
|  |  |  |  |  |  |  | LTPA | OPA | Total PA | Walking/daily PA | Commuting/transport | Domestic activity | School activity | Not specified | LPA | MPA | VPA | MVPA | Not specified | |
| Treuth et al. (2005) USA [144] | Pregnancy, parenthood | Prospective longitudinal ~ 1 yr. follow-up | | Total sample: n = 63 [F: 100%]  Low BMI group: n = 17 [F: 100%]  Normal BMI group: n = 34 [F: 100%]  High BMI group: n = 12 [F: 100%] | 18-40 [30.7 ± 4.1] 30.8 ± 3.9 30.3 ± 4.3 31.2 ± 4.5 | Q: mod. MLTPAQ | ● | ● | ● | ● |  | ● |  |  |  |  |  |  | ● | |
| ***Residence-related events/transitions*** |  |  | |  |  |  |  |  |  |  |  |  |  |  |  |  |  |  |  | |
| Regan et al. (2016) Canada [146] | Moving from community to retirement living | Prospective longitudinal 3-5 mo. follow-up | | n = 12 [F: 41.7%] | 73-87 [84.7 ± 4.0] | Q: CHAMPS AQ Accelerometry  (7 days) | ● |  | ● |  |  | ● |  |  | ● | ● | ● |  |  | |
| **LTPA** = leisure-time physical activity; **OPA** = occupational physical activity; **LPA** = light physical activity; **MPA** = moderate physical activity; **VPA** = vigorous physical activity; **MVPA** moderate-to-vigorous physical activity; **n.r.** = not reported; **Q** = Questionnaire; **EPAQ2** = EPIC-Norfolk Physical Activity Questionnaire; **PAQ-C** = Physical Activity Questionnaire for Older Children; **MAQ-A** = Modifiable Activity Questionnaire for Adolescents; **MAQ** = Modifiable Activity Questionnaire; **FPAQ** = Flemish Physical Activity Questionnaire; **CAAL** = Child/Adolescent Activity Log; **HLPLP II** = Health-Promoting Lifestyle Profile II; **GLTEQ** = Godin Leisure-Time Exercise Questionnaire; **Past Year LTPAQ** = Past Year Leisure-Time Physical Activity Questionnaire; **MESA PAQ** = The Multi-Ethnic Study of Atherosclerosis Physical Activity Questionnaire; **LAPAQ** = LASA (Longitudinal Aging Study Amsterdam) Physical Activity Questionnaire; **EPIC PAQ** = European Prospective Investigation into Cancer and Nutrition Study Physical Activity Questionnaire; **GPAQ** = Global Physical Activity Questionnaire; **HILDA Survey** = Household, Income and Labour Dynamics in Australia Survey; **PASE** = Physical Activity Scale for the Elderly; **CHAMPS AQ** = Community Healthy Activities Model Program for Seniors Activity Questionnaire; **PAR** = Physical Activity Recall; **SQUASH** = Short Questionnaire to Assess Health-Enhancing Physical Activity; **MARCA** = The Multimedia Activity Recall for Children and Adults; **MLTPAQ** = Minnesota Leisure-Time Physical Activity Questionnaire; **IPAQ** = International Physical Activity Questionnaire; **ABAKQ** = Adolescent Behaviour, Attitude, Knowledge Questionnaire | | | | | | | | | | | | | | | | | | | |  |

1. We report on sample size and gender distribution (F: female) [↑](#footnote-ref-1)
2. Age range, mean and standard deviation are reported as available. [↑](#footnote-ref-2)
3. Cf. Page AS, Cooper AR, Griew P, Davis L, Hillsdon M. Independent mobility in relation to weekday physical activity in children aged 10-11 years: The PEACH Project. Int J Behav Nutr Phy. 2009;6(2):1-9. [↑](#footnote-ref-3)
4. Age at retirement [↑](#footnote-ref-4)
