# Supplementary material for: The impact of life events and transitions on physical activity: A scoping review
Source: PLoS One. 2020 Jun 22;15(6):e0234794. doi: 10.1371/journal.pone.0234794 (PMC7307727; doi:10.1371/journal.pone.0234794)
Supplement: S2 Table — (DOCX) [file pone.0234794.s002.docx]

**S2 Table.** **Studies using the same samples**

| **Study Sample** | **Study (Citation No.)** |
| --- | --- |
| **ALSWH** | 40, 70, 71 |
| **MESA** | 92, 93 |
| **PEACH project** | 46, 50 |
| **WHI study** | 126, 130 |
| **SU.VI.MAX cohort** | 99, 106 |
| **HRS** | 89, 94, 114 |
| **FPSS^[[1]](#footnote-1)^** | 105, 116, 123 |
| **Sample of Flemish children** | 47, 48 |
| **ALSWH** = Australian Longitudinal Study on Women’s Health; **MESA** = Multi-Ethnic Study of Atherosclerosis; **PEACH** = Personal and Environmental Associations with Children’s Health project; **WHI** = Women’s Health Initiative study; **SU.VI.MAX** = Supplémentation en Vitamines et Minéraux Antioxydants cohort; **HRS** = Health and Retirement Study; **FPSS** = Finnish Public Sector Study | |

1. While Clark et al. [116], Josefsson et al. [123], and Stenholm et al. [105] were drawing on the same sample, all of them assessed different life events and transitions. [↑](#footnote-ref-1)
